# Comparing sequence and structure of falcipains and human homologs at prodomain and catalytic active site for malarial peptide based inhibitor design

**DOI:** 10.1186/s12936-019-2790-2

**Published:** 2019-05-03

**Authors:** Thommas Mutemi Musyoka, Joyce Njoki Njuguna, Özlem Tastan Bishop

**Affiliations:** Research Unit in Bioinformatics (RUBi), Department of Biochemistry and Microbiology, Rhodes University, P.O. Box 94, Grahamstown, 6140 South Africa

**Keywords:** Cysteine protease, Falcipain, Zymogen, Prodomain inhibitory segment, Homology modelling, Binding affinity

## Abstract

**Background:**

Falcipains are major cysteine proteases of *Plasmodium falciparum* involved in haemoglobin degradation and remain attractive anti-malarial drug targets. Several inhibitors against these proteases have been identified, yet none of them has been approved for malaria treatment. Other *Plasmodium* species also possess highly homologous proteins to falcipains. For selective therapeutic targeting, identification of sequence and structure differences with homologous human cathepsins is necessary. The substrate processing activity of these proteins is tightly controlled via a prodomain segment occluding the active site which is chopped under low pH conditions exposing the catalytic site. Current work characterizes these proteases to identify residues mediating the prodomain regulatory function for the design of peptide based anti-malarial inhibitors.

**Methods:**

Sequence and structure variations between prodomain regions of plasmodial proteins and human cathepsins were determined using in silico approaches. Additionally, evolutionary clustering of these proteins was evaluated using phylogenetic analysis. High quality partial zymogen protein structures were modelled using homology modelling and residue interaction analysis performed between the prodomain segment and mature domain to identify key interacting residues between these two domains. The resulting information was used to determine short peptide sequences which could mimic the inherent regulatory function of the prodomain regions. Through flexible docking, the binding affinity of proposed peptides on the proteins studied was evaluated.

**Results:**

Sequence, evolutionary and motif analyses showed important differences between plasmodial and human proteins. Residue interaction analysis identified important residues crucial for maintaining prodomain integrity across the different proteins as well as the pro-segment responsible for inhibitory mechanism. Binding affinity of suggested peptides was highly dependent on their residue composition and length.

**Conclusions:**

Despite the conserved structural and catalytic mechanism between human cathepsins and plasmodial proteases, current work revealed significant differences between the two protein groups which may provide valuable information for selective anti-malarial inhibitor development. Part of this study aimed to design peptide inhibitors based on endogenous inhibitory portions of protease prodomains as a novel aspect. Even though peptide inhibitors may not be practical solutions to malaria at this stage, the approach followed and results offer a promising means to find new malarial inhibitors.

**Electronic supplementary material:**

The online version of this article (10.1186/s12936-019-2790-2) contains supplementary material, which is available to authorized users.

## Background

Malaria, caused by parasites from the genus *Plasmodium* and transmitted to human by a female anopheles mosquito bite, remains a major public health menace with an estimated annual rate of 0.45 million fatalities [1]. Parallel to evolving mosquito resistant to insecticides [1–4], continuously emerging resistant strains of parasite to current drugs [5–8] present an immense challenge for the eradication of malaria. A recent study promisingly showed that pre-existing resistance may not be a major problem for novel target anti-malarial candidates, and fast-killing compounds may result in a slower onset of clinical resistance [9]. Hence, the identification and development of alternative anti-malarial inhibitors with novel mode of action against new as well as known drug targets is crucial.

Proteases are considered as good parasitic drug targets and details are presented in a number of articles [10–16]. Cysteine proteases have a central role in *Plasmodium* parasites during haemoglobin degradation [17, 18], tissue and cellular invasion [19], activation of pro-enzymes [20, 21], immunoevasion and egression [11, 21, 22]. Red blood cell (RBC) invasion and rupturing processes as well as intermediate events involving haemoglobin metabolism are characterized by increased proteolytic activity. During the asexual intraerythrocytic stage, *Plasmodium* parasites degrade nearly 75% of host RBC haemoglobin [23, 24] to acquire nutrients as they lack a de novo amino acid biosynthetic pathway. By this process, they can acquire all their amino acid requirements necessary for growth and multiplication with an exception of isoleucine which is exogenously imported as it is absent in human haemoglobin [10, 25, 26]. Haemoglobin degradation is an intricate and efficient multistage protein catabolic process occurring inside the acidic food vacuole [18, 27].

This study focuses on a subgroup of papain-like Clan CA plasmodial cysteine proteases, namely falcipains (FPs) of *Plasmodium falciparum* and their homologs. *Plasmodium falciparum* has four FPs; FP-1, FP-2, FP-2’ and FP-3. FP-1 is the most conserved protease among the four proteases, and its role in parasite entry into RBCs is yet to be resolved. Although its inhibition using specific peptidyl epoxides blocked erythrocyte invasion by merozoites [28], FP-1 gene disruption in blood stage parasites does not affect their growth [29, 30]. Despite its biological function remaining uncertain, FP-2′ is biochemically similar to FP-2 and shares 99% sequence identity [22, 31]. FP-2 (FP-2′) and FP-3 share 68% sequence identity and are the major cysteine proteases involved in haemoglobin degradation in the parasite [32–35]. Expression of these proteins during the blood stage by plasmodia is strictly regulated in a site-specific and time-dependent manner [28, 36, 37]. These haemoglobinases have differential expression timing during the trophozoite stage: the early phase is characterized by FP-2 abundance while FP-3 is abundant at the late stages [17, 22]. It was shown that targeted disruption of FP-2 gene in *Plasmodium* results in accumulation of undigested haemoglobin in the food vacuole and its enlargement [17], therefore, the protein can be considered as a promising drug target [38, 39]. On the other hand, inhibiting individual proteases might not be essential due to redundancy in the haemoglobin digestion stage [10], hence any inhibitor design for FPs should consider blocking the activity of both FP-2 and FP-3. The importance of FP-2 as a drug target was also indicated in a recent study in which FP-2 polymorphisms were shown that are associated with artemisinin resistance [40].

**Fig. 1:**
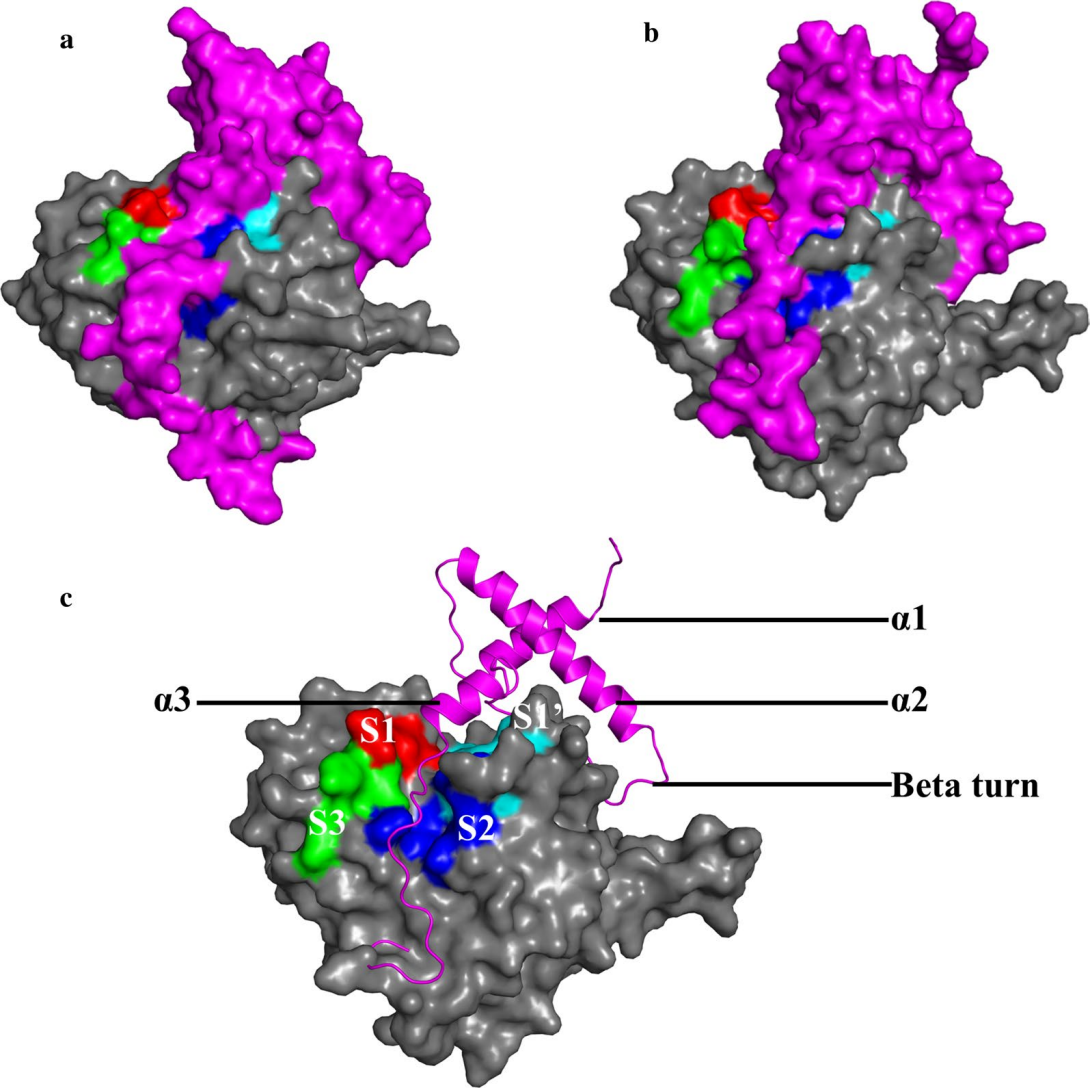
Clan CA cysteine protease zymogen prodomain-catalytic domain interaction modes. Surface representation of **a** human Cat-K and **b** FP-2. **c** FP-2 prodomain structural elements (pink; in cartoon representation) interacting with the S1 (red), S2 (blue), S3 (green) and S1′ (cyan) subsites of the catalytic domain

Other *Plasmodium* species also express proteins highly homologous to FP-2 and FP-3 [41–44]. These include vivapains (vivapain 2 [VP-2] and vivapain 3 [VP-3]), knowlesipains (knowlesipain 2 [KP-2] and knowlesipain 3 [KP-3]), berghepain 2 [BP-2], chabaupain 2 [CP-2] and yoelipain 2 [YP-2] from *Plasmodium vivax*, *Plasmodium knowlesi, Plasmodium berghei, Plasmodium chabaudi* and *Plasmodium yoelii,* respectively. All these proteins are related both in sequence and function to the papain-like class of enzymes including human cathepsins. The plasmodial proteases have, however, unusual features compared to the human ones including, much longer prodomains and specific inserts in the catalytic domain—a “nose” (~ 17 amino acids) and an “arm” (~ 14 amino acids) [37, 45, 46]. In native environment, cysteine proteases are regulated either by their prodomain (zymogen form) or by other endogenous macromolecules like cystatins [47, 48] and chagasin [49]. During erythrocyte entry, *P. falciparum* secrete falstatin, a potent picomolar inhibitor of both FP-2 and FP-3 thus regulating the activity of these proteases on important surface proteins required for invasion [19, 48]. In the zymogen form (Fig. 1), a part of the prodomain flips over the active pocket and its subsites located on the catalytic domain [50], blocking its enzyme activity [51]. The acidic environment within a food vacuole (*Plasmodium*) or lysosome (humans) triggers prodomain cleavage thus activating the catalytic domain [52, 53].

The literature comprises a large number of inhibitors against FPs derived from both experimental and computational approaches. The identified inhibitors, which are either synthetic or from natural sources, fall into three main categories: peptide-based [31, 54–56], non-peptidic [50, 57–61]; and peptidomimetic [58, 62, 63]. Majority of the peptidic and peptidomimetic inhibitors have shown activity against FPs at nanomolar concentrations. So far, the strongest potency ranging from nanomolar to picomolar concentrations has been reported from a series of 2-pyrimidine cabornitriles derivatives [58]. Application of computer assisted drug design (CADD) approaches as well as virtual screening strategies have also been utilized to identify novel non-peptidic FP compounds [57, 64]. Molecular dynamics approaches as well as binding free energy calculations have also been employed to decipher the atomic interaction details and stability of protein–ligand complexes [50, 57]. Hitherto, none of these inhibitors has been approved as an anti-malarial drug as they have limited selectivity against host cathepsins, homologs to the parasites proteases. To overcome this, distinctive features between these two classes of proteins must be determined.

The current work utilizes in silico approaches to characterize FP-2 and FP-3 and their homologs from other *Plasmodium* species as well as human homologs (cathepsins) to identify sequence, physicochemical and structure differences that can be exploited for peptide-based anti-malarial drug development. Although the two protein classes share high similarity, important differences that can be essential for inhibitor selectivity exist [50, 65]. The main aim of this study is to elucidate the inhibitory mechanism of plasmodial prodomain region responsible for endogenous regulation of the catalytic domain, information which may be useful in the design of novel peptide-based inhibitors. For this purpose, using domain–domain interaction approaches, specific hot spot residues critical for the mediation of the prodomain inhibitory effect were identified. To further identify a potential peptide segment, which could strongly bind to the plasmodial catalytic domains and mimic the native prodomain inhibitory effect, five short peptide sequences based on the identified hot spot residues were suggested. Flexible docking of these peptides against the catalytic domains identified a short 13-mer oligopeptide with preferential binding towards plasmodial proteases. This oligopeptide could be a starting platform for the development and testing of novel peptide based anti-malarial therapies against plasmodial cysteine proteases. It is known that the use of peptides for treatment of malaria faces a myriad of challenges mainly due to low permeability, metabolic instability, short half-life, low oral bioavailability and limited residence time in tissues [66, 67]. Nonetheless, several in vitro and in silico strategies have been established to enhance peptide developability as drugs [68, 69]. Currently, the use of nanotechnology for site specific drug delivery systems to increase bioavailability continues to be explored and may soon offer a breakthrough in the application of anti-malarial peptides [70–72].

## Methods

**Fig. 2:**
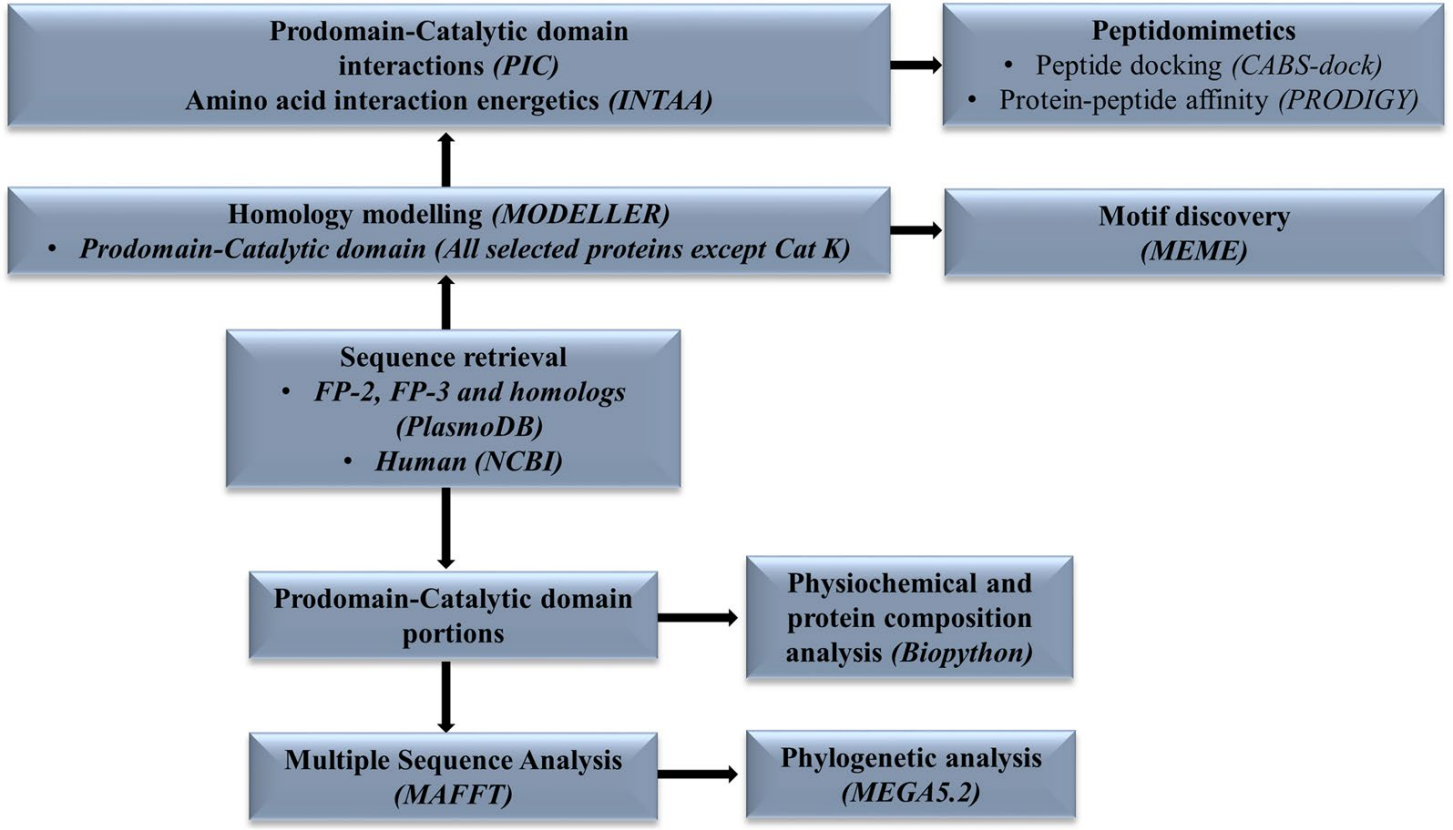
A graphical workflow of the methods and tools (in brackets) used in sequence and structural analysis of FP-2, FP-3 and their homologs

A workflow consisting of the different methods, tools and databases used in this study is shown in Fig. 2. Unless otherwise indicated, amino acid numbering is based on individual protein full length as listed in Additional file 1.

### Sequence retrieval and multiple sequence alignment

Using FP-2 (PF3D7_1115700) and FP-3 (PF3D7_1115400) as query sequences, seven plasmodial protein homologs together with three human homologs (Table 1) were retrieved from the PlasmoDB version 9.31 [73] and NCBI [74] databases, respectively as described earlier [50]. A pronounced feature present in the cathepsin L (Cat-L) like plasmodial proteases is the presence of an N-terminal signalling (non-structural) peptide sequence (~ 150 amino acids), which is responsible for targeting them into the food vacuole. For each of the plasmodial proteins, this segment was chopped off, and the remaining prodomain portion-catalytic domain saved into a Fasta file (Additional file 2). As guided by the partial zymogen complex crystal structure of Cat-K [PDB: 1BY8], ~ 21 amino acids (N-terminal) were also chopped off from the human cathepsin prodomain sequences. Together, these sequences were used in the rest of the study, and are referred as “partial zymogen” or “prodomain-catalytic domain” sequences interchangeably in the manuscript. Position details of the prodomain and catalytic portions per protein are listed in Additional file 1. To determine the conservation of the prodomain-catalytic portion, multiple sequence alignment (MSA) was performed using PROfile Multiple Alignment with predicted Local Structures and 3D constraints (PROMALS3D) web server [75] with default parameters except PSI-BLAST Expect value which was adjusted to 0.0001, and the alignment output visualized using JalView [76].

**Table 1:** Details of all protein sequences retrieved from PlasmoDB and NCBI databases

Source organism	Host	Accession number	Common name (abbreviation)	aa	% SI
*P. falciparum (Pf)*	Human	PF3D7_1115700	Falcipain-2 (FP-2)	484	QS
		PF3D7_1115400	Falcipain-3 (FP-3)	492	QS
*P. vivax (Pv)*		PVX_091415	Vivapain-2 (VP-2)	487	56^a^
		PVX_091410	Vivapain-3 (VP-3)	493	55^b^
*P. knowlesi (Pk)*	Human/monkey	PKH_091250	Knowlesipain-2 (KP-2)	495	53^a^
		PVX-091260	Knowlesipain-3 (KP-3)	479	58^b^
*P. yoelii (Py)*	Rodents	PY00783	Yoelipain-2 (YP-2)	472	47^a^
*P. chabaudi (Pc)*		PCHAS_091190	Chabaupain-2 (CP-2)	471	48^b^
*P. berghei (Pb)*		PBANKA_093240	Berghepain-2 (BP-2)	470	50^a^
*H. sapiens*	–	NP_000387.1	Cathepsin-K (Cat-K)	329	34^a^
		AAA66974.1	Cathepsin-L (Cat-L)	333	33^a^
		AAC37592.1	Cathepsin-S (Cat-S)	331	32^a^

Percentage sequence identity (SI) is calculated based on the partial zymogen of query sequence (QS) and that of corresponding homolog

^a^FP-2 homolog, ^b^FP-3 homolog

### Phylogenetic inference

Using Molecular Evolutionary Genetic Analysis (MEGA) version 5.2 software [77], the evolutionary relationship of plasmodial proteases and human cathepsins was evaluated with the following preferences; Maximum Likelihood (statistical method) and Nearest-Neighbor-Interchange (NNI) as the tree inference option. A total of 48 amino acid substitution models were calculated for both complete (100%) and partial (95%) deletion and the best three models based on Bayesian Information Criterion (BIC) were selected (Additional file 3). For each selected model, the corresponding gamma (G) evolutionary distance correction value was selected to build different phylogenetic trees and comparison was made to determine robustness of dendrogram construction process. *Toxoplasma gondii* Cat-L [NCBI accession number: ABY58967.1] was included in the tree calculations as outgroup.

### Physicochemical properties

Using an ad hoc Python and Biopython script, the amino acid composition and physicochemical properties, namely molecular weight (Mr), isoelectric point (p*I*), aromaticity, instability index, aliphatic index and grand average of hydropathy index (GRAVY) of the proteins were determined.

### Motif analysis

Multiple Em for Motif Elicitation (MEME) standalone suite version 4.10.2 [78] was used to identify the composition and distribution of protein motifs within partial zymogen sequences. A Fasta file (Additional file 2) containing sequence information of the different proteins was parsed to MEME software with analysis preferences set as; -nostatus –time 18,000 –maxsize 16,000 –mod zoops –nmotifs X –minw 6 –maxw 50. The variable X (a whole number from 1) was varied until no more unique motifs were assessable as determined by Motif Alignment Search Tool (MAST) [79]. A heat map showing motif distribution was generated using an in house Python script. PyMOL was used to map the different motifs onto the protein structures (The PyMOL Molecular Graphics System, Version 1.6.0.0 Schrödinger, LLC).

### Homology modelling and structure validation

MODELLER version 9.18 [80] was used to build homology models of the inhibitor complex of all proteins except for Cat-K which has already a crystal structure. Using a combination of templates, high quality prodomain-catalytic domain complexes of the plasmodial proteases as well as cathepsins (Cat-L and Cat-S) were calculated by MODELLER with refinement set to very slow. Additional file 4 shows the details of templates selected for each protein model. For the plasmodial proteases, the crystallographic structure of procathepsin L1 from *Fasciola hepatica* [PDB: 2O6X] was used as it had the highest similarity with most target sequences (30–38%) and high resolution of 1.40 Å. However, it lacked the arm (β-hairpin) region while the nose residues were missing. To overcome these challenges, Cat-K [PDB: 1BY8] together with FP-2 [PDB: 2OUL] (for FP-2, VP-2, KP-2, BP-2 and YP-2) and FP-3 [3BWK] (for FP-3, VP-3, KP-3 and CP-2) were additionally used. For Cat-L and Cat-S, only two templates were used [PDB: 1BY8 and 2O6X]. For each protein, 100 models were calculated and ranked according to normalized discrete optimized protein energy (Z-DOPE) score [81]. The top three models per protein were further validated using ProSA [82], Verify3D [83], QMEAN [84] and PROCHECK [85] and the best quality model selected.

### Prodomain-catalytic domain interaction studies and short inhibitor peptide design

To determine the prodomain inhibitory mechanism, residue interactions between prodomain and catalytic domain of plasmodial and human partial zymogen complexes were evaluated using the Protein Interaction Calculator (PIC) web server [86]. The interaction energy of identified residues was evaluated using the amino acid interaction (INTAA) web server [87]. PyMOL was used to visualize the resulting interactions. For each protein, prodomain segment interacting with the catalytic domain’s active pocket residues was identified and extracted into a Fasta file. From the interaction energies, residues within these inhibitory segments forming strong contacts with subsite residues were identified. Based on the identified hot spot residues, the next objective was to design short peptide(s) exhibiting the native prodomain effect whilst showing selectivity on human cathepsins. The conservation of prodomain inhibitory segments for all the proteins, and separately of only the plasmodial proteases, was determined using WebLogo server [88]. Peptides of varying lengths and composition based on amino acid conservation forming contacts with subsite residues were proposed. In order to evaluate the interaction of selected peptides on the catalytic domains, the prodomain segments of all proteins were chopped using PyMOL. Blind docking simulation runs of selected peptides were then performed on these sets of catalytic domains by CABS-dock protein-peptide docking tool [89] using the default parameters. To confirm the reliability of the results, docking experiments were repeated using catalytic domains of the same proteins that had been modelled and used in previous studies [50]. Binding affinity (ΔG) and dissociation constant (K_d_) for each protein-peptide complex was then evaluated using PROtein binDIng enerGY prediction (PRODIGY) web server [90].

## Results and discussion

**Fig. 3:**
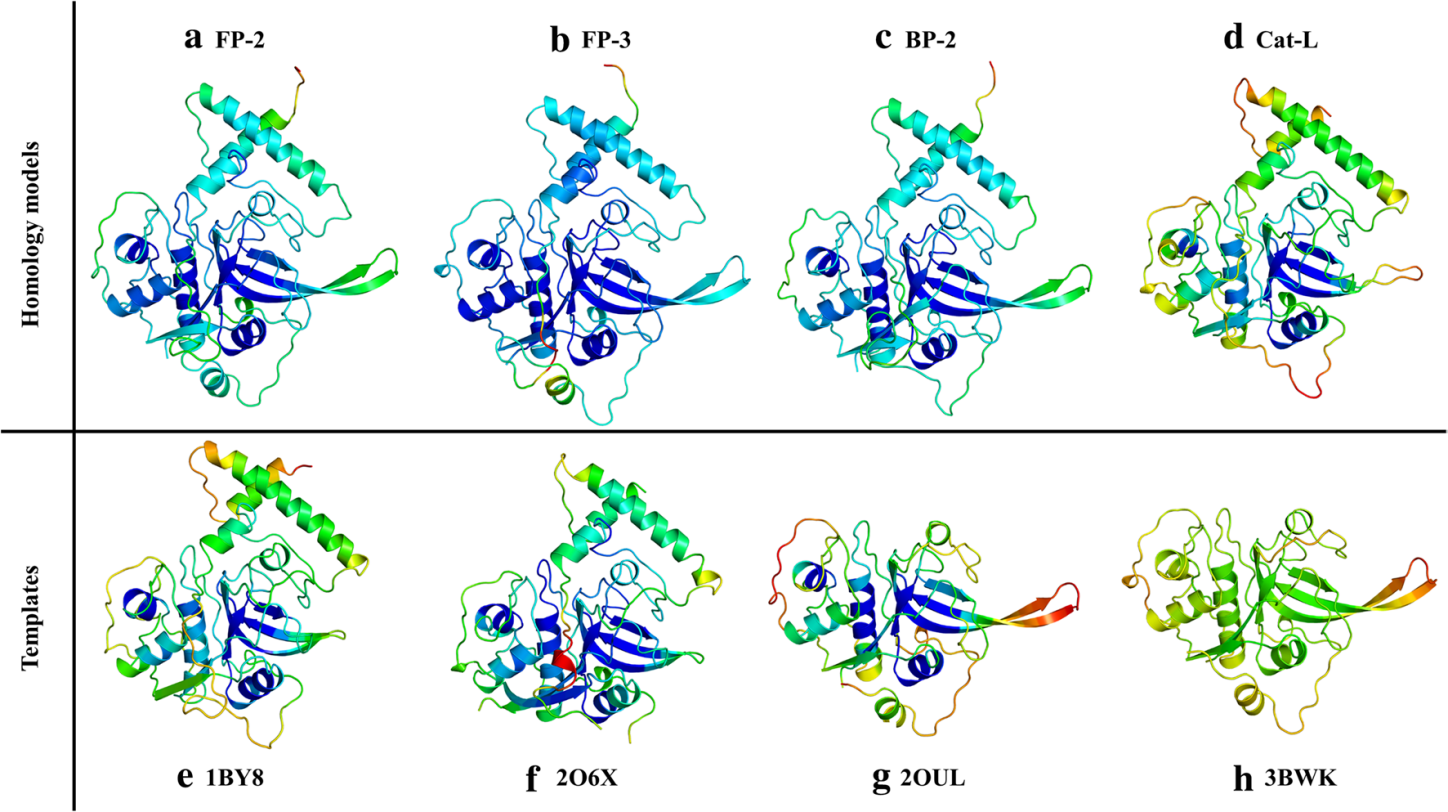
Homology models of different plasmodial proteases and human Cat-L together with the templates used in homology modelling. Colour code ranges from blue (accurate modelling) to red (poorly modelled regions)

This work is presented in two main parts: The first part analyses the proteins of interest in sequence level via physicochemical properties calculations, MSA, phylogenetic tree calculations and motif analysis with the aim of understanding the general differences between plasmodial proteins and human cathepsins. Part 2 starts to use the sequence differences identified in Part 1 at structural level with further analysis on residues that are involved in the regulation of the catalytic domain per protein with an aim of designing short peptides which could mimic the prodomain segment inhibitory mechanism. While Part 1 relies on sequence information, Part 2 requires good quality structural information. Here, 3D structures of partial zymogens are calculated via homology modelling. Accuracy of these models is checked with a range of validation tools that gave consistently high quality scores for the selected models (Table 2). QMEAN results showed that only small portions of the loop regions in Cat-L, Cat-S, and CP-2 were of poor quality, while the majority of the prodomain-catalytic core regions in all of the proteins was accurate (Fig. 3 and Additional file 5). As these loop regions were far from the catalytic pocket, the resulting models were considered acceptable for further analysis.

**Table 2:** Homology model quality validation scores of partial zymogen complexes using different assessment tools

Protein	Z-DOPE	Verify3D	ProSA	QMEAN	RAMACHANDRAN (%)		
					Favoured	Allowed	Disallowed
FP-2	− 1.05	78.48	− 8.27	0.69	88.90	10.50	0.60
FP-3	− 0.94	84.64	− 7.84	0.67	89.70	10.30	0.00
VP-2	− 0.64	81.27	− 6.94	0.62	85.40	13.90	0.70
VP-3	− 0.74	79.58	− 7.31	0.70	88.60	11.40	0.00
KP-2	− 0.63	85.89	− 7.23	0.62	89.30	9.70	1.00
KP-3	− 0.92	90.63	− 7.88	0.63	86.10	13.90	0.00
BP-2	− 0.62	86.85	− 7.75	0.63	86.60	12.40	1.00
CP-2	− 0.60	84.45	− 7.02	0.63	83.90	13.80	2.30
YP-2	− 0.42	75.54	− 7.28	0.62	84.90	14.40	0.70
Cat-L	− 1.47	87.38	− 7.94	0.87	89.80	9.80	0.40
Cat-S	− 1.61	85.39	− 8.57	0.79	89.20	10.40	0.20
1BY8	*	85.16	− 8.62	0.78	65.80	34.20	0.00
2O6X	*	94.77	− 7.00	0.77	90.50	9.50	0.00
2OUL	*	98.13	− 7.90	0.75	88.10	11.20	0.70
3BWK	*	93.51	− 7.35	0.65	86.10	13.30	0.60

* Template

### Both plasmodial and human cathepsins have similar physicochemical properties

Protein function is largely governed by its structure, amino acid composition as well as its environment. Despite the low sequence identity between the two subclasses (cathepsins and plasmodial proteases), physicochemical analysis revealed that they have similar aromaticity and grand average hydropathy (GRAVY) values indicating that both groups of proteins are hydrophilic (Table 3). With an exception of CP-2, all the other proteins have an instability index score of ≤ 40 and thus can be considered as being stable in test-tube environment [91]. Interestingly, there is no significant difference between the aromaticity, GRAVY and instability index scores of partial zymogen complex and individual catalytic domains either. However, significant differences exist in the molecular weight and isoelectric point (p*I*). Plasmodial partial zymogens have higher molecular weight than that of human cathepsins, as they have longer sequences (two additional structural catalytic domain inserts and longer prodomains). A key factor that controls the functioning of cysteine proteases is pH of the milieu in which they are found. All the plasmodial prodomain-catalytic complexes and Cat-L have a slightly acidic p*I* of 5.66 ± 0.37 with their catalytic domains exhibiting lower p*I*. The other cathepsins have basic p*I* for both their partial zymogen complexes and catalytic domains. This difference in p*I* profiles might explain the localization aspects of these proteins where the plasmodial proteases and Cat-L are found in acidic food vacuoles and lysosomes, respectively while the remaining cathepsins are predominantly found in extracellular matrix.

**Table 3:** A summary of physicochemical properties of FP-2 and FP-3 and homologs partial zymogen sequences

Protein	Aromaticity	GRAVY	Instability index	Mwgt^a^		p*I*^a^	
				Complex	Catalytic	Complex	Catalytic
FP-2	0.12	− 0.59	40.31	38,021.06	27,176.69	6.50	4.94
FP-3	0.14	− 0.53	33.90	38,029.91	27,348.62	5.47	4.72
VP-2	0.14	− 0.40	23.78	37,941.11	27,388.93	5.49	4.74
VP-3	0.14	− 0.47	28.92	38,405.76	28,088.95	5.60	5.00
KP-2	0.14	− 0.54	29.55	38,583.83	27,947.73	5.69	4.93
KP-3	0.14	− 0.51	23.16	38,284.39	27,685.36	5.89	5.13
BP-2	0.14	− 0.52	39.73	38,014.94	27,367.90	5.44	4.77
CP-2	0.13	− 0.47	52.14	37,883.92	27,140.51	5.14	4.54
YP-2	0.14	− 0.46	40.05	38,120.25	27,454.06	5.66	4.78
Cat-K	0.10	− 0.62	33.12	34,566.05	23,495.47	8.83	8.92
Cat-L	0.13	− 0.70	38.17	35,074.20	24,298.86	5.33	4.64
Cat-S	0.12	− 0.56	37.20	34,986.63	23,963.97	8.44	7.64

^a^Significant variation between partial zymogen and catalytic sequence

### Plasmodial clan CA proteases and human cathepsins exhibit separate evolutionary clustering

**Fig. 4:**
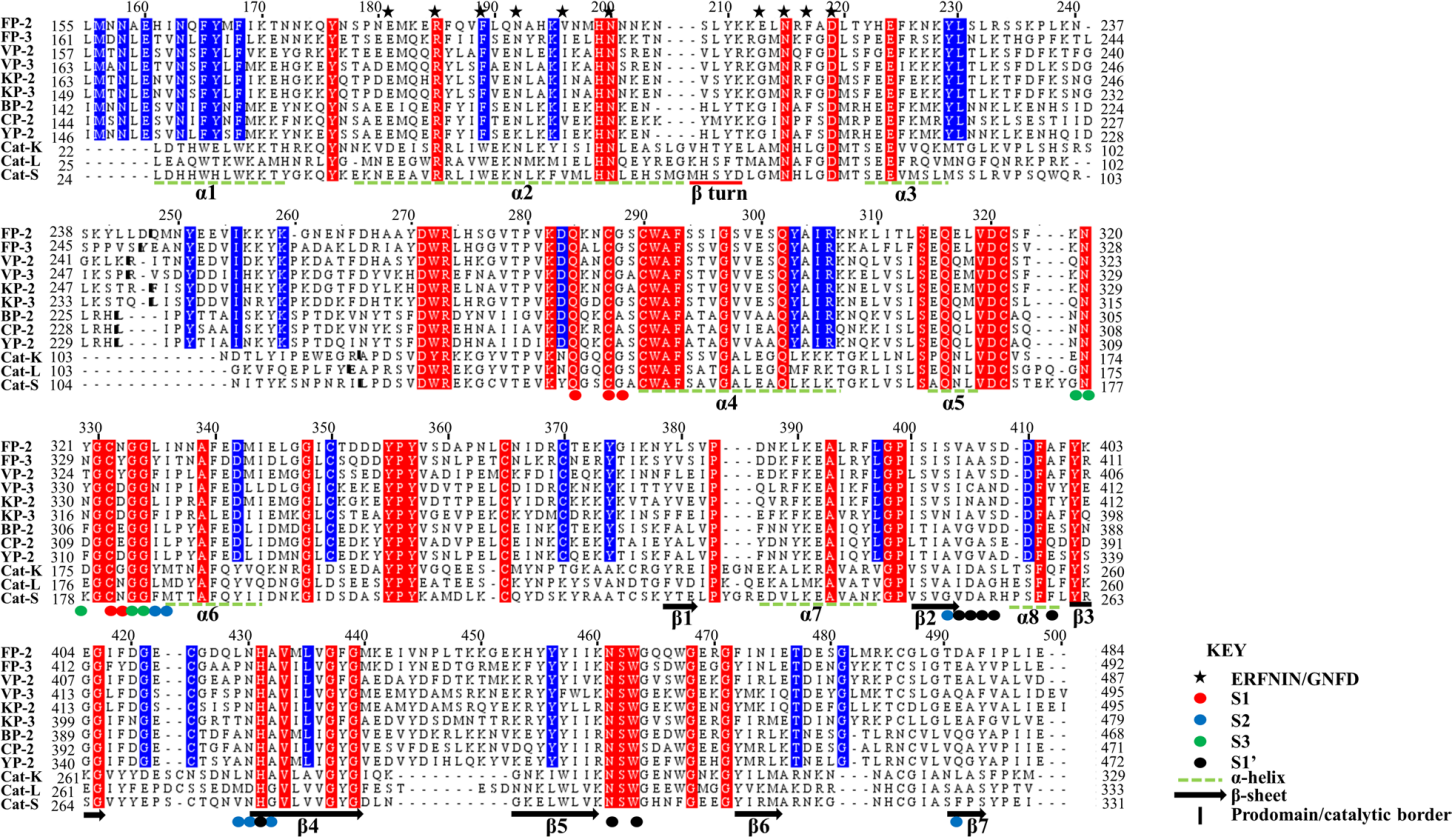
Structural-based multiple sequence alignment of FP-2, FP-3 and homologs prodomain-catalytic domains. Actual residue numbering per protein is given on the side, and the top numbering is based on partial zymogen alignment. The papain family characteristic prodomain ERFNIN and GNFD motif residues are indicated with an asterisk. Bold short lines depict the prodomain-catalytic domain border. Dashed green lines indicate the position of α-helix and arrows β-sheet structural elements. Fully conserved residues in all the proteins are marked with red while residues only conserved in plasmodial proteases with blue. Position of subsite residues is shown with filled circles (Red = S1, Blue = S2, Green = S3 and S1′ = black)

In addition to the previous findings for catalytic domain conservation discussed comprehensively in [50], current MSA identified two highly conserved ERFNIN and GNFD motifs, which are located in the α2-helix and the adjacent downstream loop region between β turn and α3-helix, respectively (Figs. 1 and 4). Despite the highly conserved nature of the ERFNIN motif across all the plasmodial proteins studied, FP-2 and CP-2 have Val residue in the place of Ile196 (numbering based on FP-2). In the human cathepsins, the motif’s Phe190 (FP-2 numbering) is replaced by a Trp, a more hydrophobic residue. Using site-directed mutagenesis, Kreusch et al. identified two additional conserved Trp residues in human Cat-L (position 29 and 32 in Cat-L full length protein) which together with the highly conserved motifs (ERFNIN and GNFD) are important in the stability of the partial zymogen complex [92]. In plasmodial proteases, conservative substitution occurs on these two residues whereby they are replaced by less hydrophobic Phe residues (position 165 and 169 in FP-2). The contribution of these amino acid variations will be further discussed in the “*Prodomain regulatory effect mediated by α3 helix hydrophobic interactions with subsites S2 and S1’ residues*” section. MSA result also revealed that cathepsins have a three amino acid insert in the α2 helix between the ERFNIN/GNFD motifs which is absent in the plasmodial proteases, and its importance is yet to be reported.

**Fig. 5:**
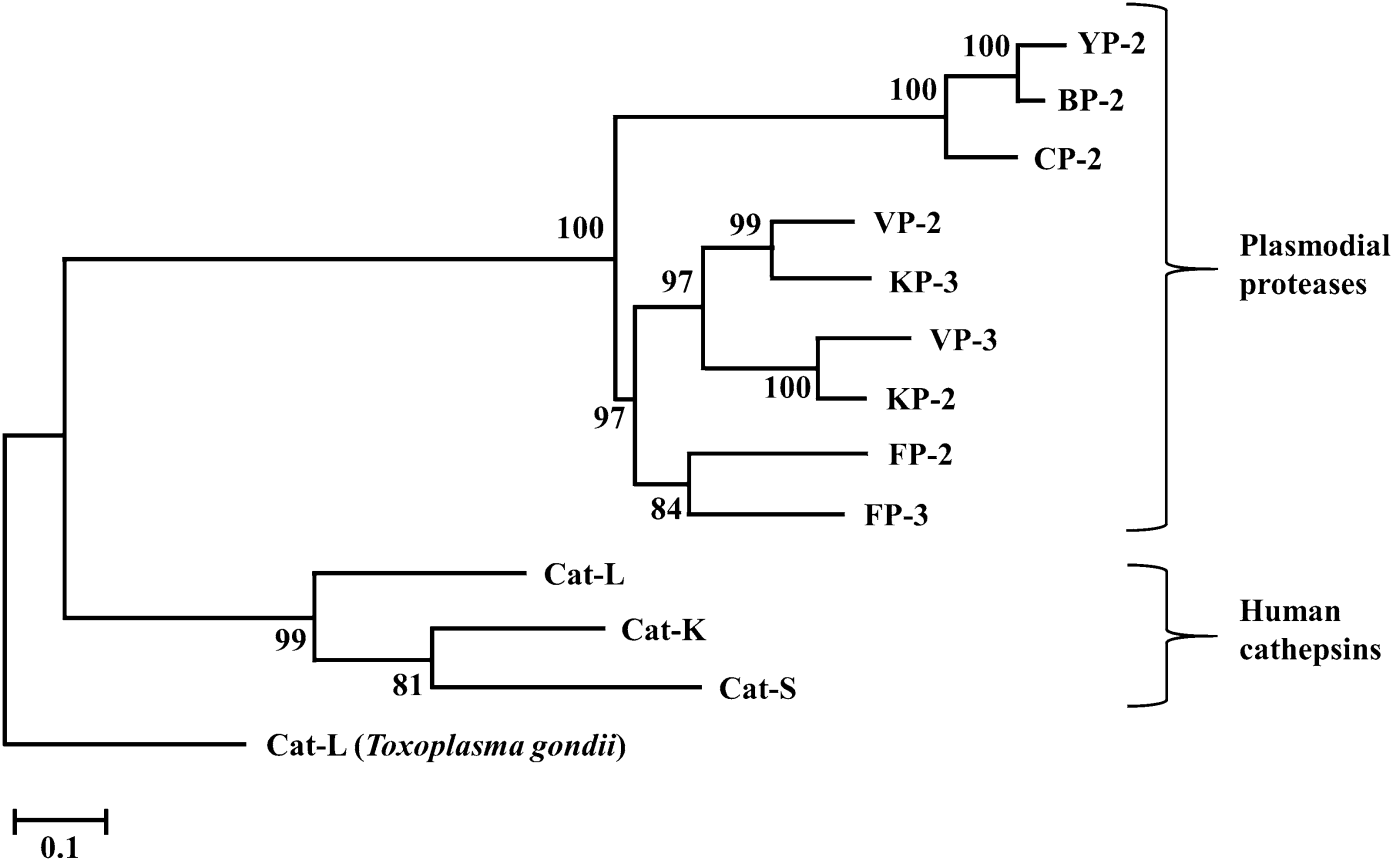
A phylogenetic tree of plasmodial and human FP-3, and FP-3 homologs prodomain-catalytic protein sequences using MEGA5.2.2. The evolutionary history was inferred by using the Maximum Likelihood method based on the Whelan and Goldman model (WAG) model with a γ discrete distribution (+G) parameter of 2.4 and an evolutionary invariable ([+I]) of 0.1. All positions with gaps were completely removed (100% deletion) and bootstrap value set at 1000. The scale bar represents the number of amino acid substitutions per site. *Toxoplasma gondii* CAT-L is used as the outgroup

Phylogenetic analysis using partial zymogen sequences gave a distinct clustering between plasmodial proteins and human cathepsins forming two separate clades (Fig. 5). There is no notable difference in tree topology in analysis performed using the catalytic domains only. This can be explained by the observed low sequence identity in both partial zymogen (Table 1) and catalytic domain sequences between the two groups of proteins [50]. The plasmodial proteases further clustered into two main subgroups based on the host. This is attributed to the previously reported sequence variations between the human and rodent plasmodial proteases [50]. FP-2 and FP-3 forms a separate sub-group from the other human plasmodial proteases possibly due to the high sequence similarity between the two proteins. The rate of mutation accumulation appears to vary between the two classes of proteins, being slowest in the human cathepsins. All human plasmodial proteases seem to evolve at the same rate as compared to the rodent orthologs which appear to show the highest substitution rate among all the proteins.

### Plasmodial proteases have unique motifs compared to human cathepsins

**Fig. 6:**
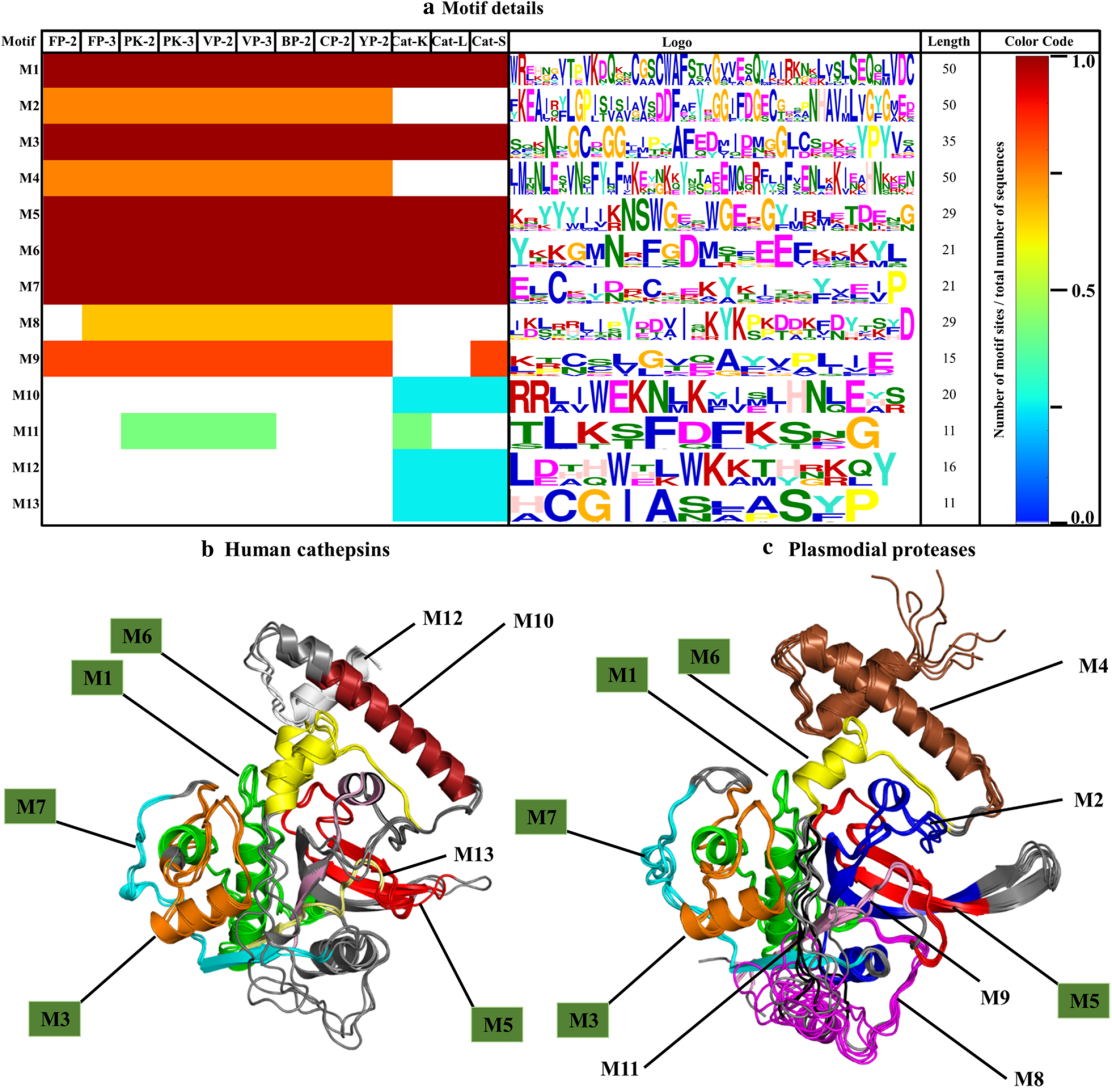
Motif analysis of plasmodial proteases and human cathepsins partial zymogen domains. **a** A heat map showing the distribution, level of conservation and information of different motifs found in plasmodial and human proteases studied. A cartoon presentation showing the location of all motifs within the prodomain-catalytic structural fold. Labelled in green boxes are motifs present in both (**b**) human cathepsins and (**c**) plasmodial proteases

Sequence motifs within proteins might be associated with a specific biological function. Thus to better understand and characterize a group of proteins, identification of common and distinguished motifs is of critical importance. A total of 13 unique motifs with varied distributions were identified in the set of proteins studied (Fig. 6a). These motifs were then mapped onto the 3D structures of partial zymogen complexes (Fig. 6b, c). Five motifs (M1, M3, M5, M6 and M7) are present in both the plasmodial and human proteases. Out of these five motifs, M1, M3, M5 and M7 are located at the catalytic domain of all proteins while M6 is at α3-helix region of the prodomain (Fig. 6b, c). Up to three motifs; M2, M4 (located in α1-helix) and M8 (nose region) are only found within the plasmodial proteases, except FP-2 lacks M8. A differential motif composition of the anterior prodomain region (α1- α3 helix) of the two classes of proteins was observed with one long motif (M4) in plasmodial proteases while human cathepsins have two (M10 and M12).

PROSITE [93] and MyHits [94] webservers were used to search for the functional importance of identified motifs. M1 (PF00112.15) is the peptidase_C1 functional site and consists of PS00139 (QQnCGSCWAfST-cysteine protease active site), PS00008 (GVvesSQ-N-myristoylation site), and PS00006 (casein kinase II phosphorylation site). M2 (PF00112) is a characteristic functional site of papain-like family cysteine proteases located at the C- terminus (α7-helix to β4), and forms part of the arm region of plasmodial proteases. M3 is located in α6-helix, and the adjacent loop regions of all the Clan CA group of enzymes have no function assigned to it. M4 (PF08246) is known as the cathepsin propeptide inhibitor domain (Inhibitor I29), and is located at α1 and α2 helixes of the N-terminus. The other motifs had no defined function assigned to them according to these webservers.

### Prodomain regulatory effect mediated by α3 helix hydrophobic interactions with subsites S2 and S1′ residues

**Fig. 7:**
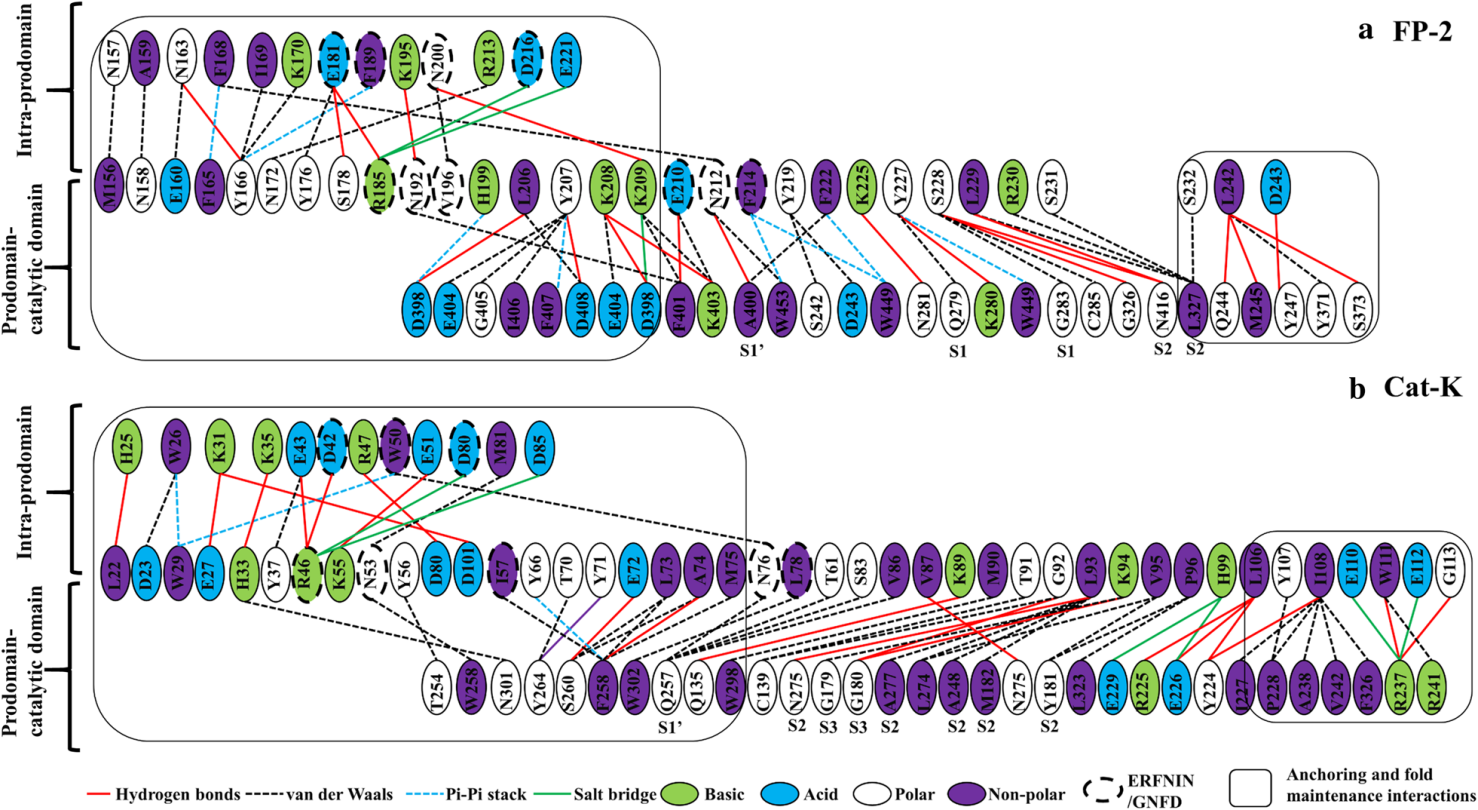
Intra-prodomain and prodomain-catalytic residue interaction network in **a** FP-2 and **b** Cat-K. For each protein full length residue numbering is used. Enclosed in black are residue interactions involved in anchoring the prodomain onto the catalytic domain with the rest being those involved in mediating the inhibitory effect. Shown in lines are the different interaction types between the prodomain and catalytic domains

Different non-canonical interactions were identified between the prodomain and catalytic domain of proteins. These included hydrophobic, cation-π, ionic, aromatic–aromatic and hydrogen bonds. In all partial zymogen complexes studied, no disulphide linkages between the two domains were observed. The main interactions exhibited are hydrophobic and hydrogen bonds, which participated either in anchoring and maintaining the folding integrity of the prodomain segment, or in mediating its inhibitory effect by interacting with subsite residues (Additional file 6). Residue interaction results revealed that prodomain anchoring residues are located on the region between α1-helix and the β-turn which interacted with β3 and part of the arm region in the catalytic domain (Figs. 4, 7). Additionally, the C-terminus of the prodomain interacts with the N-terminus of the catalytic domain and residues within α7-β3 segment (Fig. 4). For anchoring of the prodomain inhibitory segment in plasmodial proteases, a network of hydrogen bonds and hydrophobic interactions (bond order < − 10 kJ/mol) between His199-Asp398, Tyr207-Asp408 and Lys208-Glu404 (FP-2 numbering) occurred in all plasmodial proteins (Fig. 7a). These residues were only conserved in the plasmodial proteases. In the cathepsins, although such a highly conserved network is missing as observed in plasmodial proteins, the number of anchoring residues seemed to be more than in the plasmodial proteases. A strong hydrogen and hydrophobic interaction network running from the N-terminal end to the GNFD motif prodomain residues, possibly for maintaining its structural fold, was identified in all proteins (Fig. 7). In comparison with the human cathepsins, the plasmodial proteases had longer N-terminal prodomain regions (Fig. 4) harbouring a series of highly conserved residues viz. Met156, Asn158, Glu160 and Asn163 (FP-2 numbering). These residues formed a hydrophobic interaction network with bonds of the order < − 10.0 kJ/mol with neighbouring residues (Fig. 7). Two additional aromatic–aromatic interactions between Phe165-Phe168 and Tyr166-Phe189 (FP-2 numbering) in all the plasmodial proteases formed strong bonds with energies less than -20.0 kJ/mol and -10.0 kJ/mol, respectively. In human cathepsins, most of these aromatic residues (except Tyr) are substituted with Trp residues with similar bond energies, an indication of functional significance. A strong residue interaction network between the ERFNIN-GNFD motifs and other prodomain residues exists in all proteins, confirming the importance of these two motifs in maintaining its integrity and structural fold.

A previous mutagenesis study on FP-2 identified two salt bridges (Arg185-Glu221 and Glu210-Lys403) that are important in the activation of the enzyme [95]. From the residue interaction analysis, Arg185 formed a stronger salt bridge with Asp216 (− 21.2 kJ/mol) than with Glu221 (− 9.5 kJ/mol). To validate these results, Asp216 and Glu221 were independently mutated with an alanine residue and their interaction energy contribution with Arg185 was determined. A complete loss of interaction for Glu221Ala mutation was observed (0.6 kJ/mol) while Asp216Ala energy dropped by half to − 12.5 kJ/mol, an indication that the ionic pair between Arg185 and these two positions play a critical biological function. These two residues are fully conserved in all of the proteins studied here. The second predicted salt bridge by Glu210-Lys403 (FP-2 numbering) has high residue variation across all the proteins. For the charged Glu210 position in FP-2, all the other plasmodial proteases and Cat-S have a polar residue (Gly) while the other cathepsins have a non-polar residue (Ala). Most of the residues in position Lys403 (FP-2 numbering) are mainly charged except KP-3, BP-2, YP-2 and Cat-K which have a polar residue. The energetic contribution from the interactions forming this second salt bridge were insignificant in all proteins (< − 1.0 kJ/mol). However, PIC interaction results showed that position 209 in FP-2 consisted of highly conserved positively charged residue (mostly Lysine) across the other plasmodial proteases which formed strong ionic contacts with Asp398 (fully conserved in all plasmodial proteases), an indication that the second salt bridge was most likely formed by these residues. In addition, the mutagenesis study [95] also identified aromatic–aromatic interactions in FP-2 between Phe214 (of the GNFD motif), Trp449 and Trp453 to be equally important in the activation of the zymogen forms. These residues were conserved in all proteins (except Cat-K and Cat-S) and formed strong interactions, an indication that they are of functional importance as in FP-2. For Cat-K and Cat-S, strong hydrophobic interactions occur between a leucine residue in the GNFD motif (Leu78) and Trp302 (Cat-K numbering).

**Fig. 8:**
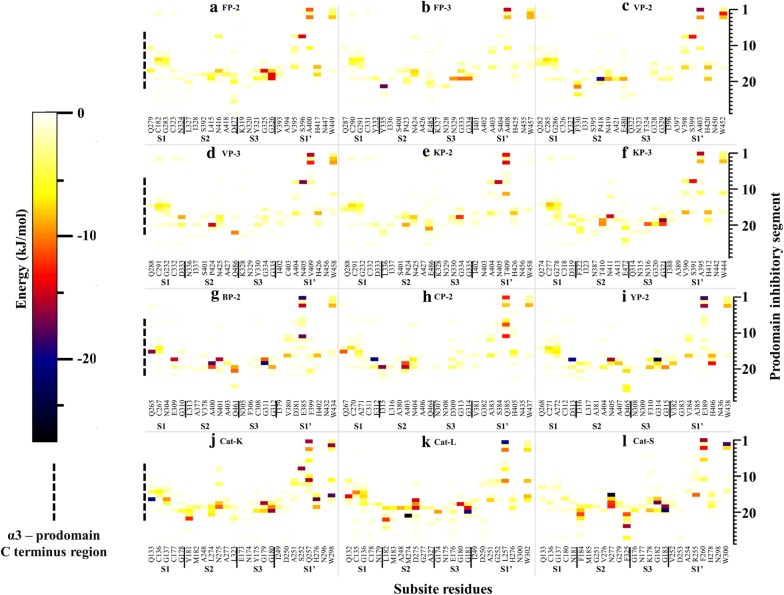
A heatmap for residue interaction energies between prodomain inhibitory segment and the catalytic subsite residues per protein. The inhibitory segment starts from the conserved Asn residue in the GNFD motif (Fig. 4)

A specific aim of this study was to determine the responsible residues that confer the prodomain with its inhibitory function. To better understand how the inhibitory prodomain per protein interacted with the four subsites of these proteins, residue energy based interaction analysis was performed. From residue interaction results, only a small portion of the prodomain (~ 22-mer) had significant contacts with individual protein subsite residues and was therefore responsible for the inhibitory effect (Fig. 8). The main residues mediating the inhibitory effect are located on the α3-helix and the downstream inter-joining C-terminus prodomain loop region, and mostly interact with subsite S2 and S1′ residues via hydrophobic interactions and hydrogen bonds (Fig. 8 and Additional file 6). This may be of significance in the development of peptide based inhibitors as previous studies have established residues forming these two subsites to be critical for the inhibitory effect and selectivity of non-peptide inhibitors [32, 50]. A common interaction profile between the prodomain inhibitory segment and the various catalytic subsite residues across all the proteins is observed (Fig. 8). For subsite S1, a limited residue contact network exists mainly with residues located at the α3-helix in all the proteases. There is a hydrophobic bond between a highly conserved Tyr226 residue (FP-2 numbering) in plasmodial proteases and the first position of subsite S1 (Figs. 4, 7a). Additional hydrogen bonding between highly conserved Lys225 and Asn281 residues (FP-2 numbering) occur in the plasmodial proteases. The C-terminal end of prodomain segment mainly exhibits contacts with S2 and S3 subsites, with human cathepsins and rodent plasmodial proteases forming stronger interactions than the human plasmodial counterparts (Fig. 8). In plasmodial proteases, a highly conserved Leu229 (FP-2 numbering) forms a hydrogen bond with subsite S2 fifth position (Asn416 [FP-2 numbering]). In all proteins, the first three prodomain inhibitory segment amino acids (part of the GNFD motif) form strong hydrophobic contacts with residues at the opening of S1′ subsite (Fig. 8). Rodent plasmodial proteases have an additional hydrogen bonding network with these residues due to the presence of a charged residue at the fifth S1′ position. From the interaction energy results, there are no observable contacts between the fourth and seventh residues (Ala215 to Thr218 [FP-2 numbering]) of the inhibitory segment in the majority of the proteins subsite residues. However, a strong hydrogen bonding and hydrophobic interaction network is formed by residues located down of the eighth residue on the prodomain inhibitory segment and various catalytic subsite residues in all the proteins. In FP-2, the network is between Ser228, Leu229, Arg230 with Leu415, Asn416 (S2), Ile406, Ala400 (S1′) and Gly325-326 (S3). Lys233 residue forms very strong ionic interactions with Asp477 (S2), a position mainly occupied by charged residues only in the human plasmodial proteases. The side-chain of Ser228 in FP-2 forms hydrogen bonding with the thiol group of catalytic Cys285. A similar interaction network in all the other plasmodial proteases was observed (Additional file 6). For the human cathepsins, these interactions seemed to be stronger especially with the third subsite S2 position. From the interaction fingerprint, residues that are key in anchoring and maintaining the stability of the prodomain as well as mediating its catalytic domain regulatory effect were identified per protein.

### Peptide inhibitory effect and selectivity dependent on composition and length

Despite their poor chemical properties, peptides remain a promising class of enzyme modulators as they are chemically diverse, highly specific and relatively safe [96, 97]. Recently, the identification of antiparasitic peptides is gaining momentum and a fully referenced database of 863 validated anti-parasite peptides from different sources has been established, of which 65% of these have anti-malarial properties [98]. Designing peptide based inhibitors requires prior understanding of how an enzyme recognizes its native peptide substrate then modifying the resulting interactions. Additionally, hot spot residues that regulate protein–protein/domain interactions may provide valuable insights. For FP-2, three peptide studies based on its prodomain-catalytic domain interaction network have already been performed. Rizzi et al. designed peptidomimetics based on the interaction information between cystatin and FP-2 [99]. Although this study focussed on FP-2, expansion to the other plasmodial proteases would have been necessary to provide additional information on the broad anti-malarial inhibitory potency of the resulting cystatin mimics. Another study by Korde et al. using a synthetic 15-mer oligopeptide based on the N-terminal extension of FP-2 partial zymogen (LMNNAEHINQFYMFI) showed that it could inhibit substrate processing activity of recombinant FP-2 in vitro [100]. Although the interaction fingerprint results using the partial zymogen complex revealed that this terminal extension was not the native inhibitory segment and was not in any way interacting with any of FP-2 catalytic domain subsite residues, their results imply that a wide array of peptides may inhibit these proteases. Further studies to establish the molecular basis of inhibition by this peptide would be necessary. Lastly, Pandey et al. expressed the whole prodomain of FP-2 together with truncated segments and evaluated their inhibitory ability against a series of papain-family cysteine proteases. At the end, they determined that a FP-2 prodomain segment (Leu127-Asp243) which included the ERFNIN and GNFD motifs had a broad inhibitory activity against FP-3, BP-2, FP-2, Cat-L, Cat-B and cruzain [101]. Considering its length and molecular mass, the therapeutic potential of this peptide is uncertain.

**Fig. 9:**
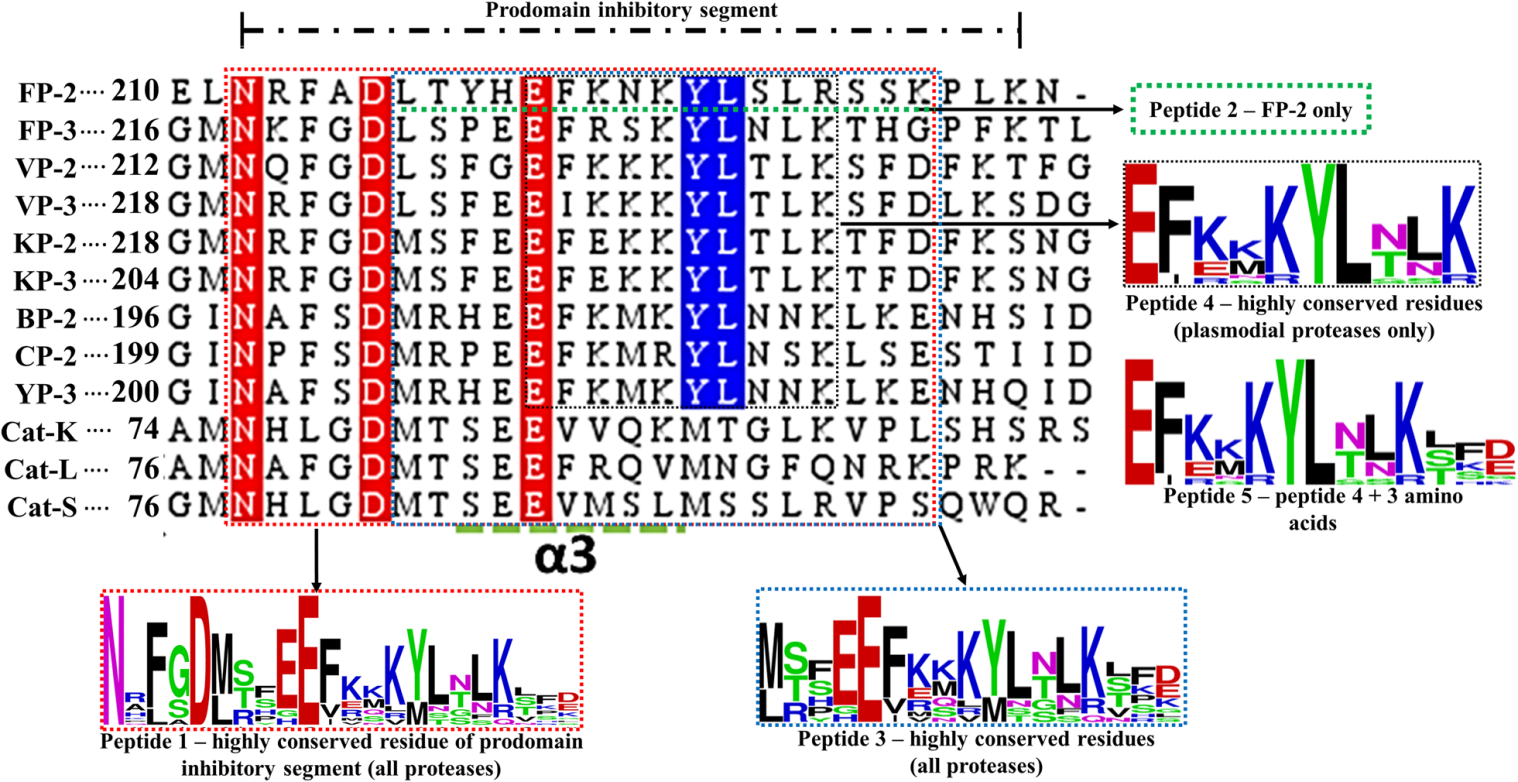
Sequence alignment of the prodomain inhibitory segment for the plasmodial and human cathepsin proteases studied. Marked sequence sections indicate the portions used to design different oligopeptides for docking studies and their conservation as determined by WebLogo server. Actual residue numbering per protein is given on the side

**Fig. 10:**
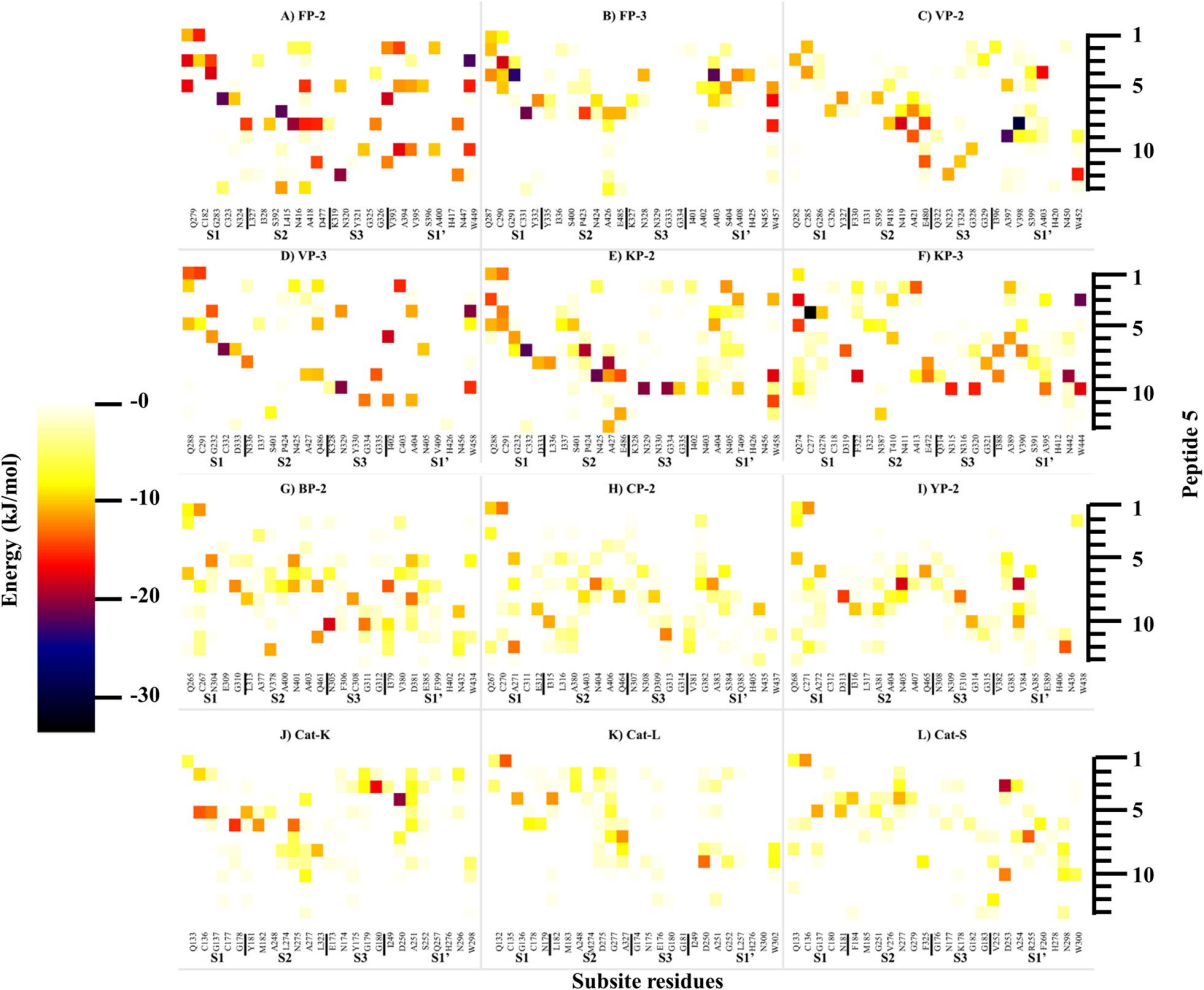
A heatmap for residue interaction energies between peptide 5 and the catalytic subsite residues per protein

**Fig. 11:**
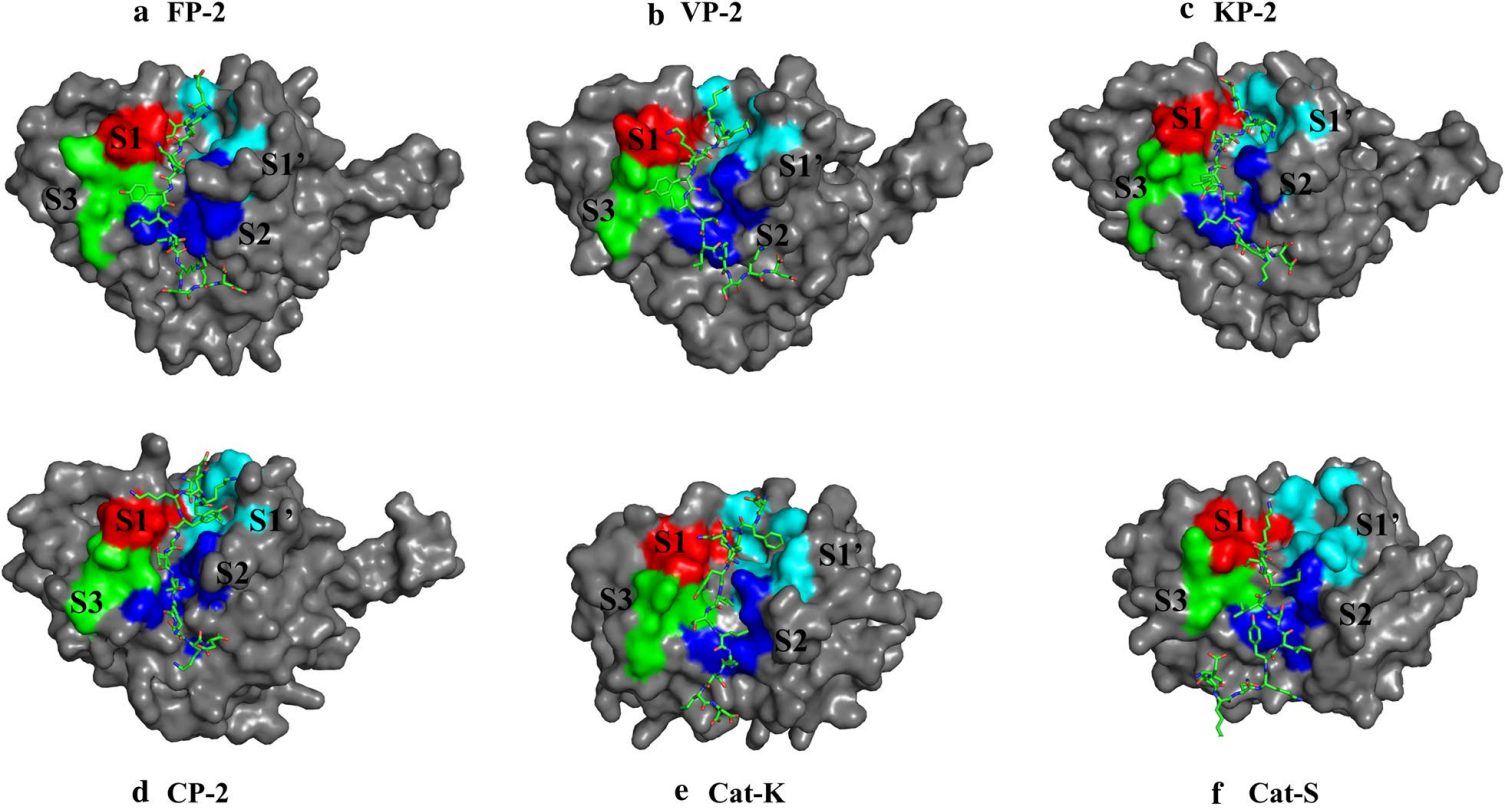
Peptide 5 binding mode with catalytic domain of various proteins (Red = S1, Blue = S2, Green = S3 and Cyan = S1′)

In the current study, peptides aimed at mimicking the inhibitory prodomain segment were designed and tested based on the identified prodomain-catalytic domain interaction fingerprint (Fig. 8). Initially, a 22-mer peptide (peptide 1 = NRFGDLSFEEFKKKYLNLKLFD) based on the conservation of the prodomain segments responsible for the inhibitory mechanism for all the proteases studied was selected for docking against the catalytic domains of individual proteins using the CABS-dock webserver (Fig. 9). CABS-dock performs blind docking simulations to identify the most probable binding site while maintaining the flexibility of the peptide ligand [89]. The ΔG of the top protein-peptide complex model per protein was then determined using the PRODIGY server. A portion of this peptide interacted with active pocket residues of individual proteins and formed complexes exhibiting high binding affinities akin to FP-2/Chagasin X-ray crystal complex [PDB: 2OUL - ΔG − 11.9, Kd= 1.9e–09] (Table 4). Despite the high predicted affinity scores with peptide 1, no differential binding was observed with the human cathepsins. An interaction analysis between the peptide and catalytic domain of the various proteins revealed that the peptide had numerous intra-chain residue interactions which enhanced dimerization and cyclization (Additional file 7). Additionally, the peptide had very strong interactions with most of the subsite residues across all the proteins. The peptide’s N-terminus which has some of the GNFD motif residues interacted with neighbouring residues in the same chain forming a clustered end (Additional file 7), a probable reason why strong contacts with residues around subsite S1 and S1′ were observed with the N-terminal prodomain inhibitory segments. Consequently, it was decided to investigate if a shorter peptide lacking these residues would bind differently without forming the clustering observed with peptide 1. A different set of docking experiments with a peptide (peptide 2 = LTYHEFKNKYLSLRSSK) derived from the main inhibitory segment of FP-2 was performed. Despite the variation in length, peptide 2 had similar results to peptide 1 and lacked a differential binding affinity profile between the two protein classes. A previous in vitro study by Pandey et al, show that a FP-2 prodomain harbouring peptide 2 segment exhibited similar broad inhibitory activity on cruzain, Cat-B, Cat-L, FP-2, FP-3 and BP-2 [101]. However, from the energy interaction profiles, a large portion of the tested prodomain including the ERFNIN/GNFD motifs is mainly involved in anchoring it to the catalytic domain. Thus, the main inhibitory segment is much shorter and downstream of the GNFD motif. Peptide 2-YP-2 complex had the strongest binding association (− 14.3 kcal/mol) while VP-2 had the lowest (− 10.1 kcal/mol). Both peptide 1 and 2 interacted with majority of the subsite residues in all the proteins studied. With the already tested peptides exhibiting unselective high affinity binding on both human cathepsins and plasmodial proteases, additional docking experiments were performed with a different peptide derived from the most conserved residues in the same inhibitory segment as peptide2 from all proteases studied (peptide 3 = MTFEEFKQKYLTLKSKD). In some positions within the prodomain inhibitory segments across the plasmodial proteases, high residue variations were observed and there was no consensus about which residue to include in the peptide. So the physicochemical properties (polar, charged, non-polar, and hydrophobic) of the residues in positions exhibiting significant variation were taken into account. In addition, residues showing stronger interactions with the catalytic subsite residues were given preference. From the affinity results, the ΔG between peptide 3 and plasmodial proteases was significantly lower in most plasmodial proteases than with the earlier peptides. From interaction analysis, subsite S1 and S1′ had reduced interaction contacts as compared with the previous peptides studied. However, Cat-K and Cat-L had similar binding affinity values with peptide 1 and 2. Guided by the residue interaction profile of prodomain residues with subsite residues (Fig. 8), a fourth peptide (peptide 4 = EFKKKYLTLK) composed of the most conserved amino acids around α3-helix of the inhibitory segment of all plasmodial proteases was evaluated. A similar trend of non-selectivity was observed as with peptide 1, though with lower binding affinity except in Cat-K. From the binding mode analysis, the peptide had a slightly different pose on Cat-K and had interactions with subsite S2 and S1′ residues which were absent in the other proteins. This may likely explain the reduced binding affinities across the proteins. A fifth peptide, similar to peptide 4 except for its length, (EFKKKYLTLKSKD) was also evaluated. The residues in this peptide showed some conservation in the plasmodial proteases and had significant differences to the human cathepsins. Interestingly, it bound more strongly to all plasmodial proteases compared to the human cathepsins (Table 4). From interaction analysis, a differential peptide-catalytic subsite bonding network was observed between the plasmodial and human cathepsin proteins (Fig. 10 and Table 5). In addition, the human plasmodial proteins had stronger interactions compared to the rodent homologs. The peptide’s N-terminus interacted with the subsite residues between subsite S1 and S1′. A strong hydrogen bond between the peptide’s third basic Lys residue and the highly conserved acidic Glu residue of the first position of subsite 1 was observed in all the proteins. The main interactions (number and strength) occurred in S2 and S1′. Previously, a high residue variation between the plasmodial and human proteases was established in both S2 and S1′ subsites [50]. The observed interaction pattern by the peptide and the proteins could be the most probable factor for the differential affinities between the plasmodial and human cathepsins (Table 4). In most of the plasmodial proteases, peptide 5 bound with almost the same affinity as that of chagasin and FP-2 (− 11.9 kcal/mol). From the prodomain-catalytic interaction analysis (Table 5 and Fig. 10), the C-terminal end in peptide 5 interacts with the last position of S2 which consists of a charged residue (only in human plasmodial proteases) forming a strong ionic interaction (< − 15 kJ/mol) as well as other non-subsite residues thus forming a stronger complex. In most plasmodial proteases, peptide 5 formed multiple hydrogen bonds especially with S2 and S1′ subsite residues. Besides this information being important in the design of strong peptide-based inhibitors, the S2 and S1′ residues unique to the plasmodial cysteine proteases with a propensity of forming strong (hydrogen and hydrophobic) interactions may also be targeted in designing non-peptide inhibitors using CADD. Similarly, the side chains of the prodomain residues establishing these interactions may be conjugated with other compounds to form novel peptidomimetic derivatives with strong inhibitory potencies against the plasmodial cysteine proteases. Binding poses of peptide 5 and the different proteins showed that the peptide fitted within the active pocket groove and also made contacts with additional non-subsite residues (Fig. 11 and Table 5). Docking studies with previously modelled catalytic domains gave results consistent with the current models. From the motif analysis (Fig. 6), a large proportion of peptide 5 was represented in motif M6. Despite the functional annotation of motif M4 indicating it as the cathepsin propeptide inhibitor domain, a majority of its residues were predominantly involved in anchoring the prodomain. Taken together, the current study is the first to identify the most likely prodomain segment involved in regulation of cysteine proteases, and to apply information based approaches to propose a peptide with differential binding on both human and plasmodial proteases.

**Table 4:** Amino acid sequences of proposed peptides, their predicted binding affinity values (ΔG—kcalmol^−1^) and dissociation constant (K_d_) with individual catalytic domains of the different proteins studied

Protein	Peptide									
	1		2		3		4		5	
	ΔG	K_d_ (M)	ΔG	K_d_ (M)	ΔG	K_d_ (M)	ΔG	K_d_ (M)	ΔG	K_d_ (M)
FP-2	− 11.8	2.2E−09	− 11.2	8.4E−09	− 8.1	1.2E−06	− 9	2.7E−07	− 11.4	4.4E−09
FP-3	− 12.2	1.2E−09	− 10.5	2.0E−08	− 9.6	9.4E−08	− 9.7	7.8E−08	− 12.3	8.8E−10
VP-2	− 11.0	8.3E−09	− 10.1	3.8E−08	− 12.3	8.8E−10	− 9.0	2.7E−07	− 10.9	1.1E−08
VP-3	− 11.7	2.7E−09	− 11.0	8.3E−09	− 9.9	5.7E−08	− 9.2	1.7E−07	− 11.8	2.1E−09
KP-2	− 12.7	4.7E−10	− 11.8	2.1E−09	− 6.7	1.3E−05	− 8.1	1.2E−06	− 10.6	1.8E−08
KP-3	− 11.8	2.1E−09	− 12.7	5.2E−10	− 9.3	1.4E−07	− 9.2	1.9E−07	− 12.7	5.2E−10
BP-2	− 11.5	3.7E−09	− 12.2	1.2E−09	− 10.9	9.9E−09	− 7.9	1.7E−06	− 11.7	2.7E−09
CP-2	− 11.9	2.2E−09	− 11.7	2.7E−09	− 8.7	3.9E−07	− 8.3	1.1E−07	− 12.7	4.7E−10
YP-2	− 11.7	2.7E−09	− 14.3	5.5E−11	− 12.4	7.9E−10	− 10.4	2.5E−08	− 9.3	1.5E−07
Cat-K	− 13.9	6.3E−11	− 11.5	3.5E−09	− 11.9	2.0E−09	− 11.4	4.2E−09	− 8.3	1.1E−07
Cat-L	− 12.2	1.0E−09	− 11.8	5.1E−09	− 12.3	9.7E−10	− 10.2	3.5E−08	− 8.7	9.2E−07
Cat-S	− 10.4	2.4E−09	− 11.5	3.5E−09	− 12.4	7.7E−10	− 9.9	5.2E−08	− 8.6	4.8E−07

Peptide 1^NRFGDLSFEEFKKKYLNLKLFD^, 2^LTYHEFKNKYLSLRSSK^, 3^MTFEEFKQKYLTLKSKD^, 4^EFKKKYLTLK^, 5^EFKKKYLTLKSKD^

**Table 5:** Peptide 5-catalytic domain residue interaction fingerprint

Protein	Subsite				Non-subsite residues
	S1	S2	S3	S1′	
FP-2	Q279, C282, G283, C323	L327, *I328*, S392, L415, N416, A418, D477	N320, G325, G326	V395, S396, A400, *H417*, A418, N447, W449	K280, W286, R470, C285
FP-3	Q287, C290, G291, Y332	*Y335*, S400, P423, A426, E485	K327, N328	A402, A403, S404, A408, *N424*, H425, N455, W457	L289, C293, W294, N338
VP-2	Q282, G286, Y327	F330, I331, P418, *N419*, A421	Q322, N323, G328, G329	I396, A397, V398, A403, H420, N451	D282, F331, P332, Y351, E367, F389
VP-3	Q288, C291, G292, C332, D333	N336, I337, S401, P424, *N425*	G334, G335, N329	I402, C403, A404, N405, *H426*, W458	D287, K289, C294, D406, S423, S457, G459, K458, W462
KP-2	Q288, C291, G292, C332, D333	L336, I337, S401, P424, *N425*, A427, E486	N329, G333, G334	N403, A404, N405, T409, H426, N456, W458	*K289*, N290, A293, C294, W295, E382, N405, D406, S457
KP-3	Q274, C277, G278, C318, D319	F322, N387, T410, N411, *E472*	Q314, N3145, N316, G320, G321	I388, A389, V390, S391, A395, *N442*, W444	G275, S279, C280, P324, R325, E368, N387, D392, T409
BP-2	Q264, A268, C308, E309	I312, A377, *A400*, N401, A403	N304, N305, F306, G311	V378, G379, D381, H402, N432, W434	K266, A272, P314, Y334, E349, A367, I376
CP-2	Q267, C270, A271, E312	I315, L316, A380, A303, *N404*, A406	N307, N308, D309, G314	V381, G382, A383, S384, *H405*, N437	K265, W274, Q306, P337, K351
YP-2	C271, A272, D313	I316, L317, A381, A404, *N405*, A407	N308, N309, F310, G314	V382, G383, V384, A385, H406, N438	Q269, W275, F310, E353, I380
Cat-K	C136, G137	Y181, M182, A248, L274, N275, A277	G179	D250, A251, S252, H276	C139, W140, F186, Y224, F256, W302, E226
Cat-L	C135, N179	L182, A248, D275, G277	N175	I249, A251, L257, H276	P172, G224, A234, I263
Cat-S	C134, *N179*	F182, G249, V274, *N275*, F323	N175, K176, G180	R253, F258, W298	Y173, Y225, E227, P229, Y230

Italics are residues forming hydrogen bonds with the peptide

## Conclusion

The current study aimed to characterize the differences between *P. falciparum* falcipains and their plasmodial and human homologs, especially where the prodomain interacts with the catalytic domain, in order to identify key residues which could be useful in anti-malarial drug development approaches. This was done at both sequence and structure level. Through homology modelling, near native 3D partial zymogen complexes of both plasmodial and human proteases were obtained. This allowed structural characterization, thus deciphering how these segments confer their inhibitory mechanism endogenously. The main prodomain residues mediating the inhibitory effect were located in the α3-helix and the inter-joining loop region, and mostly interacted with subsite S2 and S1′ residues. Previous studies showed that residues forming these two subsites are critical in inhibitor design as they differ from human cathepsins [50, 57]. Hence, putting all the analysis together, with a continuous prodomain epitope mimicking strategy, a peptide (peptide 5) which bound selectively, i.e. more strongly on plasmodial proteases than the human ones was designed. The present approach offers a starting point which could lead to the establishment of novel anti-malarial peptide drugs aimed at mimicking the natural plasmodial protease regulatory mechanism. Despite protein-peptide docking being a complex modelling problem owing to the significant conformational changes between the peptide ligand and receptor and limited availability of accurate docking scoring functions, CABS-dock engine was able to determine the binding pockets of the studied proteins accurately in virtually all docking experiments. Accessibility of parasite infected erythrocytes by macromolecules remains a major concern for the development of peptide based anti-malarial inhibitors. A study by Farias et al. using fluorescent peptides revealed that peptides with molecular weight up to 3146 Da can permeate into the blood stage parasites [102]. All the peptides determined had a mass of below 2753 Da, with peptide 5 having 1613 Da, an indication that it would readily be available inside the parasites. Korde et al. demonstrated that a synthetic 15-mer oligopeptide of mass 1885 Da could localize into the intracellular compartments of trophozoites and schizonts inhibiting FP-2 activity [100]. Additional chemical optimization of peptide 5 could provide improved derivatives with potency and selectivity together with physicochemical properties that enhance bioavailability and stability. Bioavailability remains a major challenge in the application of peptides as anti-malarial drugs despite their selectivity and potency. To overcome this barrier, nanocarrier based technology aimed at increasing the concentration of peptide and low soluble drugs in the desired tissues and cells is currently being evaluated with promising results attained so far [71]. Thus, the full potential of peptide based anti-malarial drugs may be realized in the near future.

## Additional files

**Additional file 1.** Length, numbering and location of prodomain and catalytic portions in whole and in partial zymogen sequences.

**Additional file 2.** Amino acid sequences (prodomain-catalytic portion) of Falcipain-2 and its plasmodial homologs and human cathepsins.

**Additional file 3.** Top three phylogenetic inference models for partial (95%) and complete gap deletion (100%).

**Additional file 4.** Template selection for homology modelling per each protein.

**Additional file 5.** Homology models of different plasmodial proteases and human Cat-S.

**Additional file 6.** Prodomain (first value)-catalytic domain (second value) interaction fingerprint of key residues mediating inhibitory effect (contributing to binding energy of ≤ 5.0 kJ/mol and or interacting with subsite residues).

**Additional file 7.** Binding mode of peptide 1 on catalytic domain of FP-2 (A) and human Cat-L. Showed in broken yellow lines are intra-peptide polar contacts. Subsite S1 = red, S2 = blue, S3 = green and S1′ = cyan.

## Abbreviations

Å: Angstrom
AAI: amino acid interaction
BIC: Bayesian Information Criterion
BP-2: berghepain 2
CADD: computer assisted drug design
CP-2: chabaupain 2
Cat-K: cathepsin K
Cat-L: cathepsin L
Cat-S: cathepsin S
FPs: falcipains
FP-2: falcipain 2
FP-3: falcipain 3
GRAVY: grand average of hydropathy index
K_d_: dissociation constant
KP-2: knowlesipain 2
KP-3: knowlesipain 3
MAST: Motif Alignment Search Tool
MEGA: molecular evolutionary genetic analysis
MEME: Multiple Em for Motif Elucidation
Mr: molecular weight
MSA: multiple sequence alignment
NCBI: National Centre for Biotechnology Information
NNI: nearest-neighbour-interchange
PIC: Protein Interaction Calculator
p*I*: isoelectric point
PlasmoDB: Plasmodium genome Database
PRODIGY: PROtein binDIng enerGY prediction
PROMALS3D: PROfile Multiple Alignment with predicted Local structures and 3D constraints
PSI-BLAST: Position-Specific Iterative Basic Local Alignment Search Tool
RBC: red blood cell
VP-2: vivapain 2
VP-3: vivapain 3
YP-2: yoelipain 2
Z-DOPE: normalized discrete optimized protein energy
ΔG: binding affinity
3D: three dimensional
