# Supplementary material for: Comparing sequence and structure of falcipains and human homologs at prodomain and catalytic active site for malarial peptide based inhibitor design
Source: Malar J. 2019 May 3;18:159. doi: 10.1186/s12936-019-2790-2 (PMC6500056; doi:10.1186/s12936-019-2790-2)
Supplement: Supplementary file 1 — Additional file 1. Length, numbering and location of prodomain and catalytic portions in whole and in partial zymogen sequences. [file 12936_2019_2790_MOESM1_ESM.docx]

Additional file 1. Length, numbering and location of prodomain and catalytic portions in whole and in partial zymogen sequences.

|  | **Full length**  **protein** | **Position in full length sequence** | |
| --- | --- | --- | --- |
| **Protein** |  | **Prodomain** | **Catalytic domain** |
| FP-2 | 1-484 | 155-243 | 244-484 |
| FP-3 | 1-492 | 161-249 | 250-492 |
| VP-2 | 1-487 | 157-244 | 245-487 |
| VP-3 | 1-495 | 163-250 | 251-495 |
| KP-2 | 1-495 | 163-251 | 252-495 |
| KP-3 | 1-479 | 149-239 | 240-479 |
| BP-2 | 1-468 | 142-227 | 228-468 |
| CP-2 | 1-471 | 144-230 | 231-471 |
| YP-2 | 1-472 | 146-231 | 232-472 |
| Cat-K | 1-329 | 22-114 | 115-329 |
| Cat-L | 1-333 | 25-112 | 113-333 |
| Cat-S | 1-331 | 24-114 | 115-331 |
