# Supplementary material for: Comparing sequence and structure of falcipains and human homologs at prodomain and catalytic active site for malarial peptide based inhibitor design
Source: Malar J. 2019 May 3;18:159. doi: 10.1186/s12936-019-2790-2 (PMC6500056; doi:10.1186/s12936-019-2790-2)
Supplement: Supplementary file 2 — Additional file 2. Amino acid sequences (prodomain-catalytic portion) of Falcipain-2 and its plasmodial homologs and human cathepsins. [file 12936_2019_2790_MOESM2_ESM.docx]

Additional file 2. Amino acid sequences (prodomain-catalytic portion) of Falcipain-2 and its plasmodial homologs and human cathepsins.

**>FP2**

LMNNAEHINQFYMFIKTNNKQYNSPNEMKERFQVFLQNAHKVNMHNNNKNSLYKKELNRFADLTYHEFKNKYLSLRSSKPLKNSKYLLDQMNYEEVIKKYKGNENFDHAAYDWRLHSGVTPVKDQKNCGSCWAFSSIGSVESQYAIRKNKLITLSEQELVDCSFKNYGCNGGLINNAFEDMIELGGICTDDDYPYVSDAPNLCNIDRCTEKYGIKNYLSVPDNKLKEALRFLGPISISVAVSDDFAFYKEGIFDGECGDQLNHAVMLVGFGMKEIVNPLTKKGEKHYYYIIKNSWGQQWGERGFINIETDESGLMRKCGLGTDAFIPLIE

**>FP3**

LMDNLETVNLFYIFLKENNKKYETSEEMQKRFIIFSENYRKIELHNKKTNSLYKRGMNKFGDLSPEEFRSKYLNLKTHGPFKTLSPPVSYEANYEDVIKKYKPADAKLDRIAYDWRLHGGVTPVKDQALCGSCWAFSSVGSVESQYAIRKKALFLFSEQELVDCSVKNNGCYGGYITNAFDDMIDLGGLCSQDDYPYVSNLPETCNLKRCNERYTIKSYVSIPDDKFKEALRYLGPISISIAASDDFAFYRGGFYDGECGAAPNHAVILVGYGMKDIYNEDTGRMEKFYYYIIKNSWGSDWGEGGYINLETDENGYKKTCSIGTEAYVPLLE

**>VP2**

LMTNLESVNSFYLFVKEYGRKYKTEEEMQQRYLAFVENLEKIKAHNSRENVLYRKGMNQFGDLSFGEFKKKYLTLKSFDFKTFGGKLKRITNYEDVIDKYKPKDATFDHASYDWRLHKGVTPVKDQANCGSCWAFSTVGVVESQYAIRKNQLVSISEQQMVDCSTQNTGCYGGFIPLAFEDMIEMGGLCSSEDYPYVADIPEMCKFDICEQKYKINNFLEIPEDKFKEAIRFLGPLSVSIAVSDDFAFYRGGIFDGECGEAPNHAVILVGFGAEDAYDFDTKTMKKRYYYIVKNSWGVSWGEKGFIRLETDINGYRKPCSLGTEALVALVD

**>VP3**

LMANLETVNSFYLFMKEHGKEYSTADEMQQRYLSFAENLAKIKAHNSRENVLYRKGMNRFGDLSFEEIKKKYLTLKSFDLKSDGIKSPRVSDYDDIIHKYKPKDGTFDYVKHDWREFNAVTPVKDQKNCGACWAFSTVGVVESQYAIRKKELVSLSEQEMVDCSFKNYGCDGGNIPIAFEDLLDLGGICKEKEYPYVDVTPELCDIDRCKNKYKITTYVEIPQLRFKEAIKFLGPISVSICANDDFVYYEGGLFDGSCGFSPNHAVILVGYGMEEMYDAMSRKNEKRYYFWLKNSWGEKWGEKGYMKIQTDEYGLMKTCSLGAQAFVALIDEV

**>CP2**

IMSNLESVNIFYNFMKKFNKQYNSAEEMQERFYIFTENLKKVEKHNKEKKYMYKKGINPFSDMRPEEFKMRYLNSKLSESTIIDLRHLIPYSAAISKYKSPTDKVNYKSFDWREHNAIIAVKDQKRCASCWAFATAGVIEAQYAIRQNKKISLSEQQLVDCSQNNDGCEGGILPYAFEDLIDMGGLCEDKYYPYVADVPELCEINKCKEKYTAIEYALVPYDNYKEAIQYLGPLTIAVGASEDFQDYDGGIFDGECTGFANHAVILVGYGVESVFDESLKKNVDQYYYIIRNSWSDAWGEEGYMRLKTDESGALRNCVLVQAYVPIIE

**>KP2**

LMTNLENVNSFYLFIKEHGKKYQTPDEMQHRYLSFVENLAKINAHNNKENVSYKKGMNRFGDMSFEEFEKKYLTLKTFDFKSNGLKSTRFISYDDVIHKYKPKDGTFDYLKHDWRELNAVTPVKDQKNCGACWAFSTVGVVESQYAIRKNELVSLSEQEMVDCSFKNNGCDGGLIPRAFEDMIEMGGLCKGKEYPYVDTTPELCYIDRCKKKYKVTAYVEVPQVRFKEAIKFLGPISVSINANDDFTYYEGGLFDGSCSISPNHAVILVGYGMEAMYDAMSRQYEKRYYYLLRNSWGEKWGENGYMKIQTDEFGLLKTCDLGEEAYVALIEEI

**>KP3**

LMTNLENVNSFYLFIKEHGKKYQTPDEMQQRYLSFVENLAKINAHNNKENVSYKKGMNRFGDMSFEEFEKKYLTLKTFDFKSNGLKSTQLISYDDVINRYKPKDDKFDHTKYDWRLHRGVTPVKDQGDCGSCWAFSTVGVVESQYLIRKNELVSISEQQMVDCSLQNNGCDGGFIPRALEDIIEMKGLCSTEAYPYVGEVPEKCKYDMCDRKYKINSFFEIPEFKFKEAVRYLGPISVNIAVSDDFAFYQGGIFNGECGRTTNHAVILVGFGAEDVYDSDMNTTRKRYYYIIKNSWGVSWGERGFIRMETDINGYRKPCLLGLEAFGVLVE

**>BP2**

IMNNLESVNIFYNFMKEYNKQYNSAEEIQERFYIFSENLKKIEKHNKENHLYTKGINAFSDMRHEEFKMKYLNNKLKENHSIDLRHLIPYTTAISKYKSPTDKVNYTSFDWRDYNVIIGVKDQQKCASCWAFATAGVVAAQYAIRKNQKVSLSEQQLVDCAQNNFGCEGGILPYAFEDLIDMDGLCEDKYYPYVSNVPELCEINKCTEKYSISKFALVPFNNYKEAIQYLGPITIAVGVDDDFESYNGGIFDGECTDFANHAVMLIGYGVEEVYDKRLKKNVKEYYYIIRNSWGEDWGERGYIRLKTNESGTLRNCVLVQGYAPIIE

**>YP2**

IMNNLESVNLFYSFMKKYNKEYSSAEEMQERFYIFSEKLKKIEKHNKENHLYTKGINAFSDMRHEEFKMKYLNNKLKENHQIDLRHLIPYTIAINKYKSPTDQINYTSFDWRDHNAIIDIKDQQKCASCWAFATAGVVAAQYAIRKNQKVSLSEQQLVDCAQNNFGCDGGILPYAFEDLIDMNGLCEDKYYPYVSNLPELCEINKCQEKYTISKFALVPFNNYKEAIQYLGPITIAVGVADDFESYSGGIFDGECTSYANHAVMLIGYGVEDVYDIHLQKYVKEYYYIIRNSWGEFWGEHGYMRLKTNELGTLRNCVLVQGYAPIIE

**>Cat-K**

LDTHWELWKKTHRKQYNNKVDEISRRLIWEKNLKYISIHNLEASLGVHTYELAMNHLGDMTSEEVVQKMTGLKVPLSHSRSNDTLYIPEWEGRAPDSVDYRKKGYVTPVKNQGQCGSCWAFSSVGALEGQLKKKTGKLLNLSPQNLVDCVSENDGCGGGYMTNAFQYVQKNRGIDSEDAYPYVGQEESCMYNPTGKAAKCRGYREIPEGNEKALKRAVARVGPVSVAIDASLTSFQFYSKGVYYDESCNSDNLNHAVLAVGYGIQKGNKHWIIKNSWGENWGNKGYILMARNKNNACGIANLASFPKM

**>Cat-L**

LEAQWTKWKAMHNRLYGMNEEGWRRAVWEKNMKMIELHNQEYREGKHSFTMAMNAFGDMTSEEFRQVMNGFQNRKPRKGKVFQEPLFYEAPRSVDWREKGYVTPVKNQGQCGSCWAFSATGALEGQMFRKTGRLISLSEQNLVDCSGPQGNEGCNGGLMDYAFQYVQDNGGLDSEESYPYEATEESCKYNPKYSVANDTGFVDIPKQEKALMKAVATVGPISVAIDAGHESFLFYKEGIYFEPDCSSEDMDHGVLVVGYGFESTESDNNKYWLVKNSWGEEWGMGGYVKMAKDRRNHCGIASAASYPTV

**>Cat-S**

LDHHWHLWKKTYGKQYKEKNEEAVRRLIWEKNLKFVMLHNLEHSMGMHSYDLGMNHLGDMTSEEVMSLMSSLRVPSQWQRNITYKSNPNRILPDSVDWREKGCVTEVKYQGSCGACWAFSAVGALEAQLKLKTGKLVSLSAQNLVDCSTEKYGNKGCNGGFMTTAFQYIIDNKGIDSDASYPYKAMDLKCQYDSKYRAATCSKYTELPYGREDVLKEAVANKGPVSVGVDARHPSFFLYRSGVYYEPSCTQNVNHGVLVVGYGDLNGKEYWLVKNSWGHNFGEEGYIRMARNKGNHCGIASFPSYPEI
