# Supplementary material for: Comparing sequence and structure of falcipains and human homologs at prodomain and catalytic active site for malarial peptide based inhibitor design
Source: Malar J. 2019 May 3;18:159. doi: 10.1186/s12936-019-2790-2 (PMC6500056; doi:10.1186/s12936-019-2790-2)
Supplement: Supplementary file 3 — Additional file 3. Top three phylogenetic inference models for partial (95%) and complete gap deletion (100%). [file 12936_2019_2790_MOESM3_ESM.docx]

Additional file 3: Top three phylogenetic inference models for partial (95%) and complete gap deletion (100%)

| **Gap deletion (%)** | **Model** | **BIC** | **AICc** | **lnL** | **Invariant** | **Gamma** |
| --- | --- | --- | --- | --- | --- | --- |
| **95**  **(Partial)** | WAG+G+I | 9852.5 | 9711.4 | -4832.6 | 0.1 | 2.4 |
|  | WAG+G | 9853.3 | 9718.3 | -4837.0 | - | 1.4 |
|  | cpREV+G | 9898.7 | 9763.8 | -4859.7 | - | 1.3 |
| **100**  **(Complete)** | WAG+G+I | 9852.5 | 9711.4 | -4832.6 | 0.1 | 2.4 |
|  | WAG+G | 9853.3 | 9718.3 | -4837.0 | - | 1.4 |
|  | cpREV+G | 9898.7 | 9763.8 | -4859.7 | - | 1.3 |
