# Supplementary material for: Comparing sequence and structure of falcipains and human homologs at prodomain and catalytic active site for malarial peptide based inhibitor design
Source: Malar J. 2019 May 3;18:159. doi: 10.1186/s12936-019-2790-2 (PMC6500056; doi:10.1186/s12936-019-2790-2)
Supplement: Supplementary file 4 — Additional file 4. Template selection for homology modelling per each protein. [file 12936_2019_2790_MOESM4_ESM.docx]

Additional file 4. Template selection for homology modelling per each protein.

| **Target sequence** *(aa length)* | **Template**  **PDB ID** | **Sequence similarity (%)** | **Target-Template**  **sequence coverage** | **Aligned**  **columns** |
| --- | --- | --- | --- | --- |
| Cat-L  *(309)* | 1BY8 | 53 | 1-309 | 303 |
|  | 2O6X | 46 | 2-309 | 302 |
| Cat-S  *(308)* | 1BY8 | 57 | 1-308 | 305 |
|  | 2O6X | 45 | 2-308 | 303 |
| FP-2  *(330)* | 1BY8 | 34 | 5-329 | 301 |
|  | 2O6X | 35 | 9-330 | 295 |
|  | 2OUL | 99 | 90-330 | 241 |
| FP-3  *(332)* | 1BY8 | 37 | 5-331 | 301 |
|  | 2O6X | 34 | 9-332 | 295 |
|  | 3BWK | 100 | 90-331 | 242 |
| VP-2  *(331)* | 1BY8 | 34 | 5-330 | 301 |
|  | 2O6X | 37 | 8-330 | 295 |
|  | 2OUL | 61 | 63-331 | 240 |
| VP-3  *(333)* | 1BY8 | 35 | 5-330 | 301 |
|  | 2O6X | 35 | 8-330 | 295 |
|  | 3BWK | 56 | 90-330 | 241 |
| KP-2  *(333)* | 1BY8 | 33 | 6-330 | 300 |
|  | 2O6X | 34 | 9-333 | 297 |
|  | 2OUL | 56 | 63-331 | 240 |
| KP-3  *(331)* | 1BY8 | 37 | 5-330 | 301 |
|  | 2O6X | 37 | 9-331 | 295 |
|  | 3BWK | 60 | 90-329 | 240 |
| BP-2  *(327)* | 1BY8 | 31 | 5-326 | 300 |
|  | 2O6X | 35 | 8-327 | 295 |
|  | 2OUL | 52 | 62-327 | 239 |
| CP-2  *(328)* | 1BY8 | 31 | 7-327 | 299 |
|  | 2O6X | 35 | 8-328 | 296 |
|  | 3BWK | 49 | 90-327 | 238 |
| YP-2  *(327)* | 1BY8 | 30 | 5-326 | 299 |
|  | 2O6X | 34 | 8-327 | 294 |
|  | 2OUL | 49 | 62-327 | 239 |
