# Supplementary figures and images for: Comparing sequence and structure of falcipains and human homologs at prodomain and catalytic active site for malarial peptide based inhibitor design
Source: Malar J. 2019 May 3;18:159. doi: 10.1186/s12936-019-2790-2 (PMC6500056; doi:10.1186/s12936-019-2790-2)

Additional file 5. Homology models of different plasmodial proteases and human Cat-S.


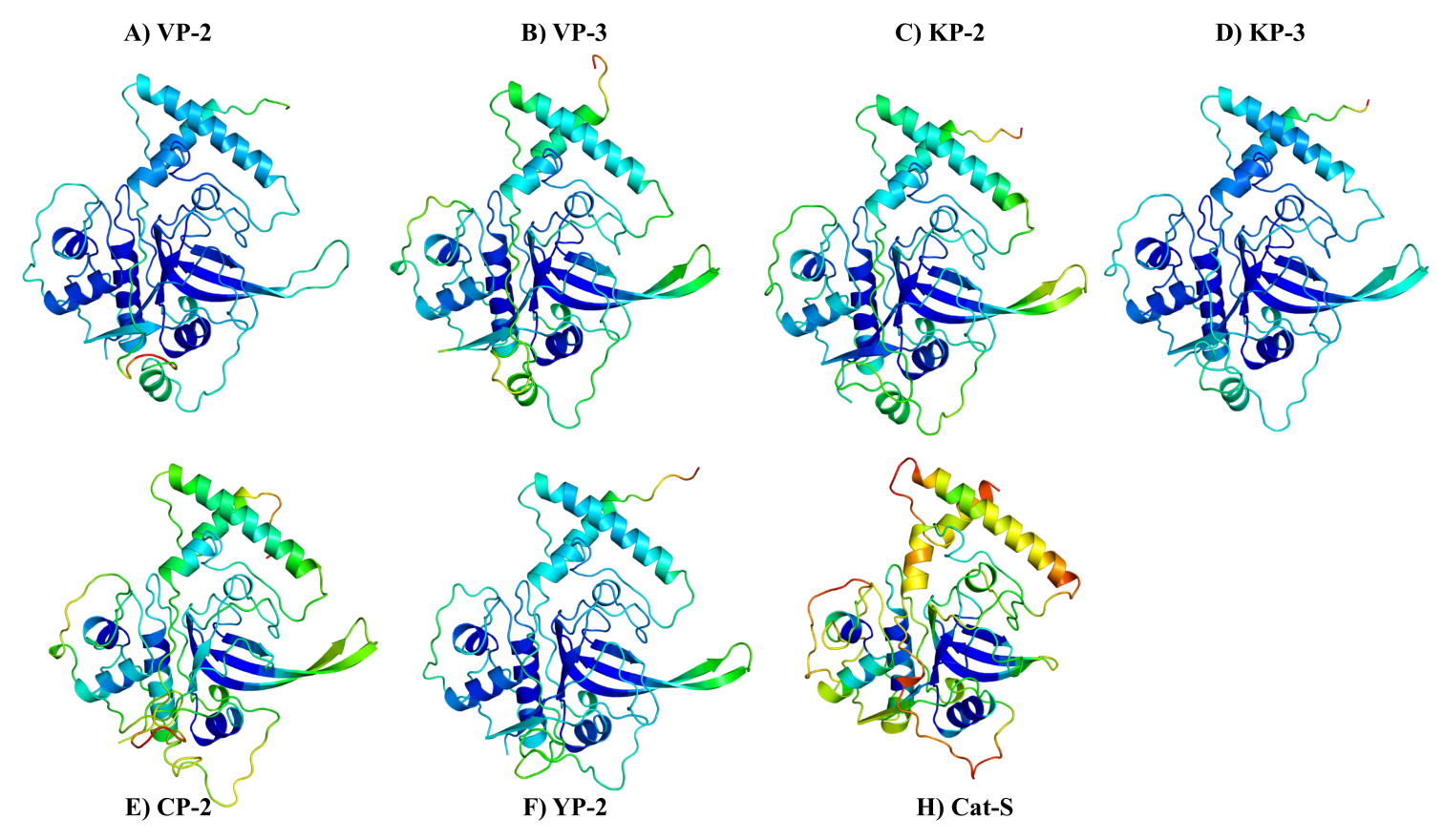

Supplement: Supplementary file 5 — Additional file 5. Homology models of different plasmodial proteases and human Cat-S. [file 12936_2019_2790_MOESM5_ESM.docx]
