# Supplementary material for: Comparing sequence and structure of falcipains and human homologs at prodomain and catalytic active site for malarial peptide based inhibitor design
Source: Malar J. 2019 May 3;18:159. doi: 10.1186/s12936-019-2790-2 (PMC6500056; doi:10.1186/s12936-019-2790-2)
Supplement: Supplementary file 6 — Additional file 6. Prodomain (first value)-catalytic domain (second value) interaction fingerprint of key residues mediating inhibitory effect (contributing to binding energy of ≤ 5.0 kJ/mol and or interacting with subsite residues). [file 12936_2019_2790_MOESM6_ESM.docx]

Additional file 6. Prodomain (first value)-catalytic domain (second value) interaction fingerprint of key residues mediating inhibitory effect (contributing to binding energy of ≤ 5.0 kJ/mol and or interacting with subsite residues).

|  | **Subsite** | | | | **Non-subsite residues** |
| --- | --- | --- | --- | --- | --- |
| **Protein** | **S1** | **S2** | **S3** | **S1’** |  |
| **FP-2** |  | L229-L327 (-1.0), L229-L415 (-5.5), L229-A418 (-5.2), **S228-N262 (-8.5)**, **L229-N416 (-2.9)**, K236-D477 (-3.1) | **S228-G326**  **(-8.4),**  **R230-G326**  **(-14.4)** | N212-A400  (-11.6)  F214-A400  (-10.1), F214-W449 (-8.6), Y219-V395 (-3.6), F222-V395 (-3.1), F222-A400 (-6.3), L227-V395 (-6.0) | L206-I406 (-24.5), Y207-Y402 (-7.9), Y207-F407 (-23.5), K208-E404 (-22.6), E210-F401 (-8.4), L242-M245 (-14.2), **L242-S373 (-8.7)**, K208-K403 (-15.7), Y226-K280 (-15.5), K209-D398 (-9.5), K225-N281 (-5.3), H199-D408 (-9.8), K209-D397 (-5.6), R213-D278 (-5.1) |
| **FP-3** | Y232-L289 (-5.3) | H238-Y335 (-18.4), T237-E485 (-4.7) | K236-G333 (-11.1), K236-N329 (-9.9) | F220-W457 (-9.1), F228-A408 (-6.1), F228-W457 (-1.5), Y232-W457 (-1.4), L233-A403 (-4.5), N234-N424 (-7.9), N218-A408 (-15.4) | I202-F409 (-7.5), Y213-Y415 (-6.0), L244-V488 (-5.2), L244-I258 (-6.8), K214-G412 (-13.3), G216-F409 (-9.7), S248-E251 (-8.1), K242-N253 (-7.4), L244-I258 (-6.4), K242-S381 (-35.3), K214-R411 (-17.5), Y232-A288 (-20.6), F241-E251 (-14.4), R215-D405 (-10.1), K219-D460 (-5.2), R215-F409 (-24.2) |
| **VP-2** |  | L231-P418 (-1.0), F234-F330 (-10.4), F236-F330 (-7.2), T230-N419 (-4.1), K232-P418 (-20.0) | K232-G329 (-3.9) | F216-A403 (-9.8), F216-W452 (-8.6), F221-V398 (-5.1), F221-A403 (-0.5), F224-V398 (-2.5), F224-A403 (-5.1), Y228-W452 (-17.8), L229-V398 (-8.1), N214-A403 (-17.1), Q215-W452 (-13.6) | I198-F404 (-8.4), L208-I409 (-21.8), Y209-F410 (-19.2), M213-F404 (-5.9), R210-G407 (-12.6), G210-F404 (-10.2), L243-N248 (-5.8), K244-E250 (-21.3), Y228-A283 (-21.5), K237-E376 (-6.5), K211-D401 (-17.6), Q215-S455 (-18.2), R245-D260 (-5.0), Y209-F410 (-19.2), |
| **VP-3** | L235-A404 (-5.6) | T236-N425 (-6.7), K238-P424 (-15.0), K238-Q486 (-4.6) | K238-Y330 (-4.0) | F222-V409 (-13.4), F222-W458 (-9.6), F227-V409 (-3.5), I230-V409 (-16.6), Y234-W458 (-1.0), N220-V409 (-15.8) | I204-Y410 (-6.7), R216-G413 (-13.9), G218-Y410 (-8.5), L214-D417 (-11.6), Y234-K289 (-19.2), K243-E382 (-5.5), R251-E354 (-9.5), K203-D406 (-8.3), R216-E412 (-25.3), K217-D406 (-5.5), K217-D407 (-9.6) |
| **KP-2** |  | L237-P424 (-50.0), T236-N425 (-8.3), K238-E486 (-4.1) |  | F222-W458 (-9.1), F230-W458 (-3.6), Y234-W458 (-10.8), L235-A404 (-5.7), N220-T409 (-14.1), R221-D287 (-9.3) | I204-Y410 (-8.2), Y215-F416 (-21.7), F222-Y410 (-5.1), K216-G413 (-13.6), G218-Y410 (-9.9), K216-E412 (-18.6), S214-L415 (-4.2), K217-D407 (-17.0), K233-N290 (-12.0), T239-E485 (-11.6), K203-D406 (-7.8), K217-D406 (-5.9) |
| **KP-3** | N206-A395 (-15.8) | L223-F322 (-4.3), L223-A413 (-3.2), F226-F322 (-4.3), F228-F322 (-2.5), T222-N411 (-14.9), L223-N411 (-1.1) | K224-G321 (-12.4), K224-N316 (-11.7), | F208-W444 (-8.7), P213-V390 (-4.8), P213-A395 (-1.0), F216-V390 (-2.9), F216-A395 (-5.2), F216-W444 (-4.6), Y220-W444 (-0.4), L221-V390 (-9.4) | I190-F396 (-8.1), Q237-F366 (-16.7), K202-G399 (-18.4), S200-N403 (-23.2), Y220-G275 (-16.0), T222-D319 (-5.4), T236-F366 (-23.0), L201-G404 (-9.7), Y201-D393 (-7.6), K219-D276 (-4.5), K189-D392 (-5.2), K203-D393 (-9.0), F228-F474 (-3.6) |
| **BP-2** |  | L217-A400 (-0.5), N214-N401 (-16.2), N215-N401 (-0.5) |  | F200-W434 (-9.0), F208-V380 (-2.7), F208-W434 (-13.4), Y212-W434 (-2.2), L213-V380 (-8.7), N198-E385 (-19.7), N214-E309 (-15.4), N215-H402 (-8.0), F200-E385 (-15.4) | L192-I391 (-24.4), Y193-F392 (-21.1), L217-V458 (-5.8), T194-G389 (-13.2), G196-S386 (-6.2), Y212-Q265 (-17.3), Y193-G394 (-5.3), K195-D383 (-12.7), K218-Y231 (-5.0) |
| **CP-2** |  | L220-A403 (-4.1), N217-N404 (-7.3), K219-A403 (-15.8), K212-N404 (-6.9) | K219-D309 (-4.2) | F203-W437 (-9.1), F211-W437 (-25.9), Y215-W437 (-0.5), L216-A383 (-6.2), N201-Q388 (-13.4) | M195-I394 (-22.1), Y196-F395 (-18.1), K198-G392 (-16.0), G199-D389 (-6.4), L197-D391 (-19.3), L197-G392 (-16.0), Y196-D386 (-10.4), M195-D396 (-7.8), H188-D386 (-6.4) |
| **YP-2** | N218-D313 (-20.9) | N218-N405 (-16.4), N219-N405 (-6.4) | K220-G315 (-10.2), N219-G315 (-0.6), K220-F310 (-8.9) | F204-W438 (-9.2), F212-V384 (-3.0), F212-W438 (-12.3), Y216-W438 (-3.2), L217-V384 (-8.6) | L196-I395 (-26.1), Y197-F396 (-23.2), T198-G393 (-15.6), G200-S390 (-5.7), N202-E389 (-19.2), D228-K243 (-23.1), Y197-G398 (-9.3), K199-D386 (-5.2), K199-D387 (-10.7), Y197-F396 (-23.2), Y216-K270 (-10.3) |
| **Cat-K** | M75-C136 (-7.0), | L93-Y181 (-2.9), L93-M182 (-4.9), L93-L274 (-7.1), L93-A277 (-5.2), V95-L274 (-7.3), V95-L323 (-1.2), P96-Y181 (-12.9), P96-L323 (-3.8), L93-N275 (-11.7), M90-Q133 (-11.2), T91-Q133 (-22.5) | **K94-G180 (-16.9)**, **G92-G180** (-4.5) | L78-W298 (-4.7), V86-W98 (-6.7), M90-W298 (-18.9), N76-Q257 (-16.5), H77-W298 (-16.9), T82-Q257 (-14.9) | Y71-Y264 (-22.2), L73-F258 (-20.8), M75-F258 (-7.5), L78-F258 (-5.1), I108-I227 (-13.5), I08-P228 (-9.6), E72-K261 (-19.8), A74-F258 (-10.8), L106-E226 (-6.1), E72-S260 (-24.4), T70-V263 (-24.3), T91-C139 (-10.1), L106-R225 (-30.0), W111-R237 (-16.8), R114-R241 (-7.0), H99-E226 (-6.2), Y71-Y264 (-22.2), M75-F258 (-7.5) |
| **Cat-L** | M92-Q132 (-14.6) | F95-G174 (-7.3), F95-M183 (-6.8), F95-A248 (-9.2), F95-M274 (-7.1), F95-A327 (-3.6), G94-D275 (-10.3), F95-D275 (-15.0) | G94-G181 (-20.3),  Q96-G181 (-20.1), N93-D275 (-15.2), N97-M294 (-27.7) | Q90-W292 (-7.6), Q90-L257 (-10.8), F88-W302 (-8.7), M92-W302 (-8.9), N78-L257 (-21.0), R89-G252 (-1.6), N93-A251 (-8.3) | M58-F258 (-7.2), I59-F258 (-8.7), F73-Y259 (-4.7), F73-Y264 (-20.2), F80-F258 (-5.5), F106-V226 (-7.3), F106-226 (-10.8), F106-P229 (-6.1), F106-A238 (-10.3), F106-V232 (-2.9), Y112-A114 (-7.7), Y112-P115 (-6.4), T74-E261 (-9.4), A76-F258 (-12.8), K104-F225 (-25.6), F106-D227 (-8.0), G94-G181 (-4.5), K104-D227 (-10.6), T74-K260 (-31.2), S72-I263 (-36.3), N97-E272 (-7.6), G103-D227 (-9.9), R98-Y185 (-9.1), E108-K237 (-8.3), F73-Y264 (-20.2) |
| **Cat-S** |  | L95-F184 (-5.3), L95-M185 (-5.4), L95-V276 (-0.2), V97-F184 (-12.3), V97-F325 (-12.0), P98-F184 (-7.5), P98-F325 (-4.9) | R96-G183 (-24.3), S94-G183 (-2.7) | L90-F260 (-14.1), L80-W290 (-7.8), M83-F260 (-8.1), V98-F260 (-5.1), L91-W300 (-3.2), M91-A254 (-0.5) N78-F260 (-16.8), H79-W300 (-19.1), S94-G137 (-1.1) | M58-P257 (-7.5), Y73-Y267 (-18.6), Y73-Y268 (-22.9), L75-L261 (-26.0), L75-Y262 (-8.1), Y107-L230 (-12.2), D74-S264 (-11.8), G76-L261 (-13.0), D74-R263 (-32.1), S72-V266 (-30.2), R96-G176 (-6.3), R103-L230 (-2.1), R103-P326 (-11.5), N104-E229 (-8.7), Y107-E240 (-17.4) |
