# Supplementary material for: Comparing sequence and structure of falcipains and human homologs at prodomain and catalytic active site for malarial peptide based inhibitor design
Source: Malar J. 2019 May 3;18:159. doi: 10.1186/s12936-019-2790-2 (PMC6500056; doi:10.1186/s12936-019-2790-2)

Additional file 7. Binding mode of peptide 1 on catalytic domain of FP-2 (A) and human Cat-L. Showed in broken yellow lines are intra-peptide polar contacts. Subsite S1 = red, S2 = blue, S3 = green and S1’ = cyan.


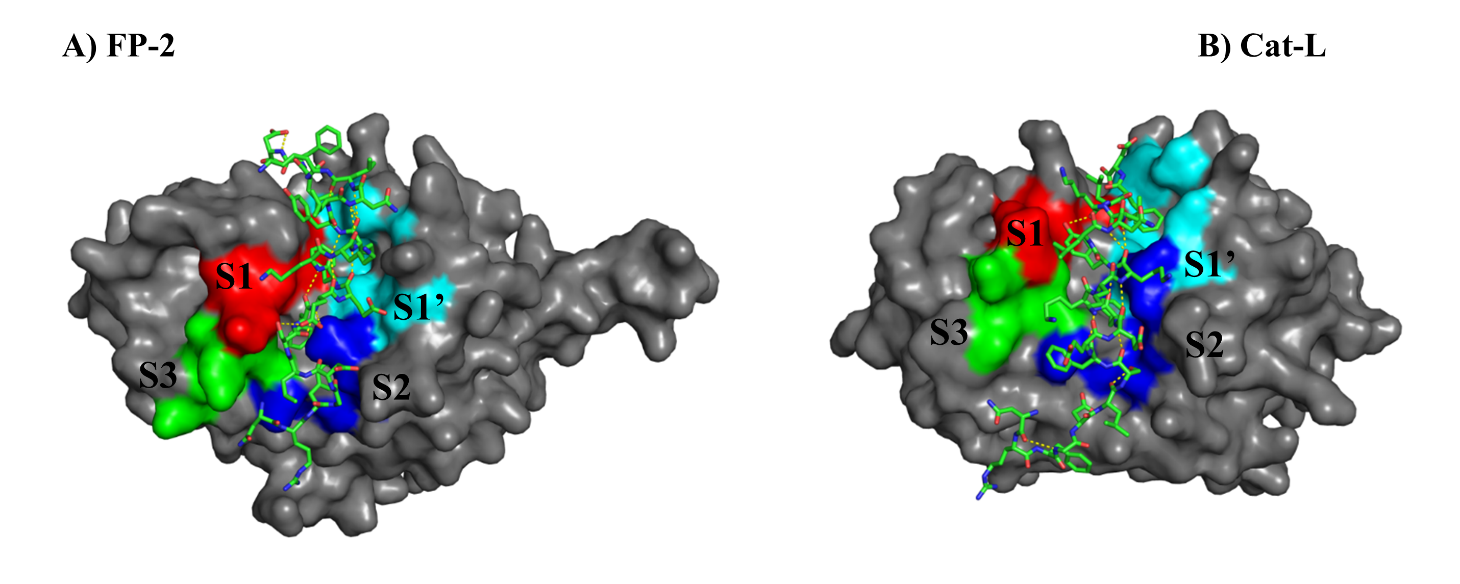

Supplement: Supplementary file 7 — Additional file 7. Binding mode of peptide 1 on catalytic domain of FP-2 (A) and human Cat-L. Showed in broken yellow lines are intra-peptide polar contacts. Subsite S1 = red, S2 = blue, S3 = green and S1′ = cyan. [file 12936_2019_2790_MOESM7_ESM.docx]
